# Climatic Variables and Virulence May Contribute to the Population Dynamics of *Pyricularia oryzae* at Local Scale

**DOI:** 10.1002/mbo3.70196

**Published:** 2026-01-14

**Authors:** Simone Bosco, Fabiano Sillo, Paola Ruffa, Lisa Bergonzi, Daniele Tenni, Pamela Abbruscato, Daniela Torello Marinoni, Marco Romani, Davide Spadaro

**Affiliations:** ^1^ Dept. Agricultural, Forest and Food Sciences (DISAFA) University of Torino Grugliasco Italy; ^2^ Interdepartmental Center for Innovation in the Agro‐environmental Sector (AGROINNOVA) Grugliasco Italy; ^3^ Institute for Sustainable Plant Protection National Research Council (CNR‐IPSP) Torino Italy; ^4^ Ente Nazionale Risi, Centro Ricerche Sul Riso Castello d'Agogna Italy; ^5^ Nuova Genetica Italiana Villa Guardia Italy

**Keywords:** genetic diversity, population structure, Pyricularia oryzae, Rice, SSR

## Abstract

Rice blast disease, caused by *Pyricularia oryzae* Cavara, is the most threatening rice pathogen in Italy. The development of resistant cultivars is a sustainable approach to mitigate yield losses. However, *P. oryzae* genomic plasticity often allows rapid adaptation to host resistance. Understanding the population structure of plant pathogens is crucial for assessing their genetic diversity and implementing durable management strategies. Despite first attempts from Piotti et al. (2005) to study the Italian *P. oryzae* population, a gap in current local *P. oryzae* genetic variability still needs to be addressed. Here, the population structure of a large set of *P. oryzae* isolates from diseased rice panicles, collected in five different Italian regions between 2011–2012 and 2020–2022, was characterized using SSR genotyping. Mating type was determined to investigate the occurrence of sexual reproduction in Italy. The integration of different cluster analyses of 200 unique multilocus genotypes allowed to identify five distinct genetic clusters. Analysis of molecular variance and of genetic divergence revealed a limited influence of geographic origin and time on population structure. A strong positive correlation was detected between climatic variables and allelic diversity in Piedmont, the most evenly sampled region in this study. The gradual disappearance over time of a genetic cluster could be linked to lower virulence on a susceptible rice cultivar. This study provides new insights into the genetic dynamics of Italian *P. oryzae* population, supporting the strategic deployment of resistance genes in rice breeding programs.

## Graphical abstract

The Italian population structure of *P. oryzae* was analyzed using SSR genotyping to identify possible determinants shaping its local genetic diversity, including spatial, temporal, environmental, and biological factors.

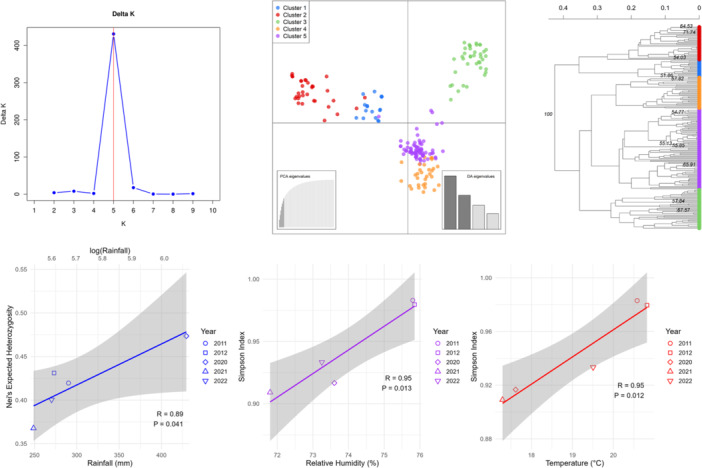

## 1 Introduction

*Pyricularia oryzae* Cavara (synonym *Magnaporthe oryzae*) is the causal agent of rice blast disease, which represents one of the main threats to rice cultivation worldwide (Savary et al. 2019). This pathogen can infect all rice aerial tissues at various phenological stages, causing spindle‐shaped necrotic lesions on leaves and nodes (leaf blast) as well as on panicle necks and branches, eventually leading to reduced grain filling (panicle blast) (Guo et al. 2024; Webster and Gunnell 1992). In Italy, the most common and lead to direct yield losses; therefore, rice blast is considered the most important and damaging disease affecting Italian rice cultivation (Bregaglio et al. 2016; Tenni et al. 2021; Titone et al. 2015).

The main disease management strategies are represented by preventive chemical control and use of resistant rice cultivars, coupled with good agronomic practices, for example, avoiding excessive nitrogen fertilization (Mongiano et al. 2021; Titone et al. 2015). Quinone outside Inhibitors represent one of the few classes of active ingredients available in the EU and the most effective against the disease. However, widespread application of single active ingredients rather than mixtures targeting multiple molecular sites is leading to resistance onset, causing the recent emergence of resistant strains in Italy as well (D'Ávila et al. 2021; Tenni et al. 2021). Genetic resistance, on the other hand, has often been reported as the most effective and environmentally friendly rice blast management method, compared to chemical control (Fukuoka et al. 2009; Miah et al. 2013). This strategy can be achieved through qualitative, with the introgression of major resistance (R) genes, or quantitative approaches, resorting to Quantitative Trait Loci, associated with partial resistance (Ashkani et al. 2016; Gou et al. 2023). According to the gene‐for‐gene relationship, plants R proteins can prevent disease occurrence by recognizing the corresponding pathogen's avirulence (AVR) effector proteins that, in turn, allow to overcome host defences in absence of a cognate R gene (Silué et al. 1992; B. Wang et al. 2017). Nowadays, more than 40 *P. oryzae* AVR genes have been identified and can be exploited for target R genes deployment in rice breeding programs (Hu et al. 2022; Selisana et al. 2017). The pathogen, however, is known for its genome plasticity, often leading to genetic resistance breakdown (Chuma et al. 2011; Hu et al. 2022). Known strategies employed by *P. oryzae* to successfully overcome host defence mechanisms are point mutations or loss of AVR genes (Hu et al. 2022; Le Naour—Vernet et al. 2023; Selisana et al. 2017), transposable elements (TE) and repeated elements‐mediated translocations, duplications or losses of virulence‐related genes (Baudin et al. 2024; Nakamoto et al. 2023; Yoshida et al. 2016) and presence of supernumerary mini‐chromosomes involved in intrachromosomal rearrangements (Kusaba et al. 2014; Langner et al. 2021).

Studying the *P. oryzae* population structure represents an important strategy to understand the pathogen's genetic diversity and, therefore, apply durable management practices. Worldwide lineages are now well established, including three major clonal populations distributed in all rice‐cultivating continents and one lineage with individuals capable of sexual reproduction mainly localized in South‐East Asia (Latorre et al. 2020; Saleh et al. 2012; Thierry et al. 2022). Genetic recombination due to sexual mating was confirmed for Asian strains in different population studies through linkage equilibrium analysis using molecular markers, the identification of female‐fertile strains and an evaluated mating type ratio (Mat1.1/Mat1.2) of 1:1 (Le et al. 2023; Qi et al. 2021; Saleh et al. 2014; Thierry et al. 2022). Population structure studies at local level with clonal lineages, needed to investigate local determinants of genetic variability, such as the effect of rice cultivar and environmental conditions on pathogen population selection, were also performed. Due to the economic importance of *P. oryzae* and its distribution worldwide, several studies using different genotyping techniques have been conducted in a range of diverse areas, including India (Sheoran et al. 2021; Yadav et al. 2019), China (Qi et al. 2021), Vietnam (Le et al. 2023), United States (Pagliaccia et al. 2018; X. Wang et al. 2017) and Brazil (D'Ávila et al. 2016; Gonçalves et al. 2016), as well as various African countries (Mutiga et al. 2017; Odjo et al. 2021; Onaga et al. 2015). In Europe, the first attempt to assess *P. oryzae* population structure in the main rice cultivating areas was conducted by Roumen et al. (1997), who detected five main lineages within a collection of 41 isolates from France, Hungary, Italy, Spain, and Portugal. Further investigation on regional population structure was conducted more than 10 years ago in Spain (Lara‐Álvarez et al. 2010) and in Italy (Piotti et al. 2005), leaving a gap into the current knowledge of *P. oryzae* genetic variability, especially in the major European country for rice production. Many of these *P. oryzae* population structure studies (D'Ávila et al. 2016; Gonçalves et al. 2016; Le et al. 2023; Odjo et al. 2021; X. Wang et al. 2017; Yadav et al. 2019) were based on microsatellite (Simple Sequence Repeats, SSR) genotyping, thanks to the development of specific SSR targeting primers optimized for population studies (Adreit et al. 2007). Molecular markers based on SSRs are commonly used for the genetic characterization of fungal plant pathogens due to their abundance in the genome, high level of polymorphism, ability to detect intra‐specific subgrouping in clonal populations, and reproducibility (Guichoux et al. 2011; Sillo 2022).

The aim of the present work was to study the Italian *P. oryzae* population structure through SSR genotyping, in order to define the current genetic variability of the pathogen and the main factors affecting its allelic diversity. Five Italian rice cultivation areas (Piedmont, Lombardy, Veneto, Emilia‐Romagna, and Sardinia) were sampled in 5 years (2011, 2012, 2020, 2021, 2022) to assess the extent to which genetic variability can be attributed to temporal and geographic factors. The influence of climatic variables on genetic diversity was also explored. Additionally, the virulence of representative strains from the inferred subpopulations was assessed to investigate biological differences among the subgroups obtained from the SSR analysis.

## 2 Materials and Methods

### 2.1 Sampling and Monoconidial Isolation

**FIGURE 1:**
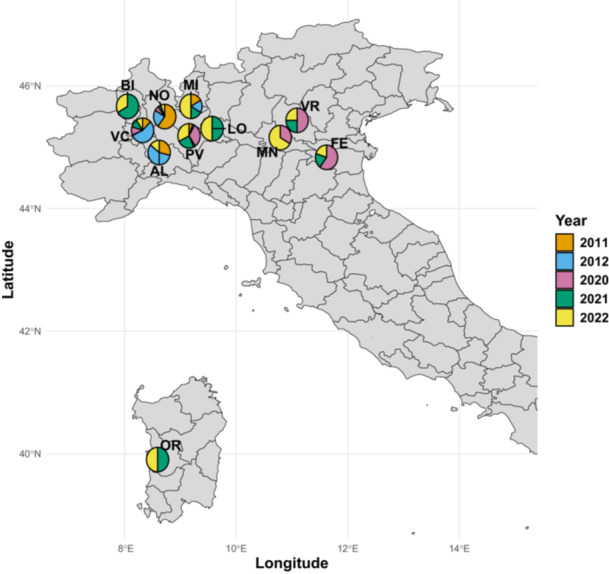
Geographical origin of 242 *P. oryzae* isolates represented as piecharts indicating the percentage of genotypes based on year of collection.

Rice panicles displaying blast symptoms were collected in years 2011, 2012, 2020, 2021, and 2022 from five different Italian regions, that is, Piedmont, Lombardy, Veneto, Emilia‐Romagna, and Sardinia (Figure 1, Table S1). The first sampling campaign was carried out in Piedmont region, mainly considering fields with elevated rice blast incidence, whereas the 2020–2022 sampling was aimed to adequately cover the main rice cultivation areas in Italy, proportionally to each region's surface area dedicated to rice cultivation. Specifically, 242 commercial fields (hereafter referred as “sites”) were sampled, including 81 in 2011, 67 in 2012, 32 in 2020, 28 in 2021, and 36 in 2022. Based on the region of origin, 185 sites were considered in Piedmont, 48 in Lombardy, 5 in Emilia‐Romagna, 4 in Veneto, and 2 in Sardinia (Table S2). For each site, 50 symptomatic panicles were collected between rice flowering stage and harvesting. In total, 242 monoconidial isolates of *P. oryzae*, one for each site, were obtained from diseased panicles as described in Tenni et al. (2021).

### 2.2 DNA Extraction, PCR Amplification and SSR Genotyping

Genomic DNA extraction from *P. oryzae* isolates was conducted using E.Z.N.A. Fungal DNA Mini Kit (Omega Bio‐tek, USA) with the addition of a mechanical disruption step with a Tissuelyser (Qiagen, Germany) at 25 Hz for 25 min. Molecular identification was performed for each isolate with species‐specific primers pfh2a (5′‐CGTCACACGTTCTTCAACC‐3′) and pfh2b (5′‐CGTTTCACGCTTCTCCG‐3′) targeting the Pot2 transposon (Terensan et al. 2022) to confirm the morphological identification. The reaction was conducted in a total volume of 25 μL containing: 2.5 μL of Buffer 10×, 2 μL of MgCl2, 1 μL of dNTPs (10 mM), 2 μL of each primer (20 mM), 0.25 μL of Taq DNA polymerase (Qiagen), and 10 ng of template DNA. Thermal cycling program consisted in an initial denaturation at 94°C for 2 min, followed by 30 cycles of denaturation at 94°C for 45 s, annealing at 55°C for 45 s and extension at 72°C for 45 s, and a final extension step at 72°C for 10 min.

A set of 16 previously developed SSR markers was selected for genotyping, including 6 developed by Adreit et al. (2007), 5 by Zheng et al. (2008), and 5 by Saleh et al. (2012). Selection of SSRs was performed based on the potential number of detectable alleles, Polymorphism Information Content (PIC), and distribution across *P. oryzae* chromosomes (Table 1). Mating type of each isolate was also determined by PCR amplification, using European reference strains from Roumen et al. (1997), all belonging to Mat1.1 idiomorph, as positive controls, with the primers developed by Tredway et al. (2003): MAT1‐1F (5′‐ATGAGAGCCTCATCAACGGCA‐3′) and MAT1‐1R (5′‐ACAGGATGTAGGCATTCGCAGGAC‐3′); MAT1‐2F (5′‐ACAAGGCAACCATCTGGACCCTG‐3′) and MAT1‐2R (5′‐CCAAAACACCGAGTGCCATCAAGC‐3′). Fifteen microliters PCR mixtures for SSR and MAT genes amplification were prepared with 1.5 μL of Buffer 10×, 0.45 μL of MgCl_2_ for SSRs, 0.3 μL of dNTPs (10 mM), 0.75 μL of each primer (10 mM), 0.15 (SSR) or 0.2 μL (MAT) of Taq DNA polymerase (Qiagen), and 10 ng of template DNA. Thermal cycling was set as follows: an initial denaturation at 94°C for 5 min, 35 (SSR) or 30 cycles (MAT) of denaturation at 94°C for 30 s, annealing at 56°C (SSR) or 60°C (MAT) for 45 s and extension at 72°C for 60 s, with a final extension step at 72°C for 15 min (SSR) or 5 min (MAT). For each mating type PCR run, a negative control (no template) and an inter‐species negative control (*Fusarium fujikuroi* strain A5, deposited at Turin University Culture Collection with code TUCC00000714) were included to confirm primer specificity. Representative MAT1‐1 amplicons (*n* = 50, 10 for each sampling year) were Sanger‐sequenced to confirm idiomorph identity. Forward SSR primers were labeled at the 5' end with either 6‐FAM, HEX, PET, or NED fluorophores to simultaneously genotype multiple regions with different size ranges. PCR products were separated using the SeqStudio™ Genetic Analyzer (Applied Biosystems‐ThermoFisher Scientific, USA), and alleles were determined with the GeneMapper™ v6 software (Applied Biosystems‐ThermoFisher Scientific) using GeneScan‐500LIZ as a size marker.

**TABLE 1:** Characteristics of the 16 microsatellite regions chosen for this study and locus‐specific metrics, including number of alleles and gene diversity, estimated using Nei's expected heterozygosity (*H*_*e*_) and Polymorphism Information Content (PIC).

Locus	Chromosome	Repeat motif	Primer sequences	Reference	Number of alleles	*H*_*e*_	PIC
pyrms37	2	CA/GT_6+_CT/GA_12_	F: ACCCTACCCCCACTCATTTC R: AGGATCAGCCAATGCCAAGT	Adreit et al. (2007)	1	‐‐‐	‐‐‐
pyrms83	2	TCA/AGT_13_	F: GTCTGCCTCGACTCCTTCAC R: GCAAAGTTGTTTGAGCAAGG	Adreit et al. (2007)	4	0.38	0.32
pyrms233	5	CAG/GTC_10_	F: TGAGATGGACCGCATGATTA R: TTGATGGCAGAGACATGAGC	Adreit et al. (2007)	3	0.37	0.26
pyrms409	4	TA/AT_23_	F: TCCCAGTACTTGCCCATCTC R: CTCCGATTCATGGCACACAC	Adreit et al. (2007)	4	0.12	0.14
pyrms427	5	AT/TA_16_	F: CTGTCACCACAACCAAGACG R: TTGCCCTGATTTGTCAGTCA	Adreit et al. (2007)	6	0.74	0.71
pyrms657	6	CA/GT_12_	F: ATCAGTCGAACCCACAAAGC R: ATGTGTGGACGAACCAGTCC	Adreit et al. (2007)	3	0.53	0.46
pyrms77b	3	CA_24_	F: AGGCTCTCTGCCTACGAAGT R: GCTTTCGGCAAGCCTAATC	Saleh et al. (2012)	3	0.3	0.31
pyrms177b	4	AC_21_	F: TCACAAGGATGGATTGCTTC R: CAGTTCGAGGTTAGCCGTTC	Saleh et al. (2012)	3	0.34	0.3
pyrms385	7	TAG_9_	F: CCTTGTTTTCCCCCTGTGTA R: TGGGAAGAAGAGACCGAAGA	Saleh et al. (2012)	3	0.17	0.16
pyrms683b	6	CTAC_18_	F: TGGGTGCAGTGCAGTTTAAT R: TGCGGCTAACTGGCAACTAT	Saleh et al. (2012)	8	0.75	0.72
pyrms685	3	TGC_12_	F: AGAACGCCAATGGAGGAAG R: GCACACCGTCGTCTATACCC	Saleh et al. (2012)	5	0.22	0.23
MGM266	6	TACC_48_	F: TGTGGTGGGTGATCTTGTTG R: ATTCCCGGCGAGAGAGATT	Zheng et al. (2008)	15	0.86	0.84
MGM288	7	TTG_14_	F: TTGTCGACGAGTGTCCAAAG R: CAGTTACCCCGTCGGTATTG	Zheng et al. (2008)	7	0.55	0.55
MGM429	5	AG_43_	F: ATTTCGTACAATCCCGATGC R: GGGTTTGCGACAATGTCTTT	Zheng et al. (2008)	14	0.9	0.89
MGM437	3	TCT_11_	F: GCCCCTCAATAGATCGTCAA R: ACTGCGGCATTTTAACCTGT	Zheng et al. (2008)	11	0.87	0.86
MGM438	4	GA_68_	F: ATCCAAAGGTGGACGTTGAG R: CAGCGGCTGATACGAAAAAT	Zheng et al. (2008)	2	0.46	0.35

Reference strains from population study by Roumen et al. (1997) of European *P. oryzae* genetic diversity (IT0010, FR0013 and SP0001, strains representative of lineage 1; IT0002, lineage 2; HN0001, lineage 3; SP0006, lineage 4; and IT0003, lineage 5), as well as additional representative strains of European diversity selected from the study by Thierry et al. (2022) isolated in 2008 (IT0046, IT0104, FR0160, HN0006, PR0009 and PR0025), were kindly provided from CIRAD (Montpellier, France) and included in the analysis. Strains with code “IT” are from Italy, “FR” from France, “SP” from Spain, “HN” from Hungary, and “PR” from Portugal.

### 2.3 Population Structure Analysis

Genetic and genotypic metrics were estimated from microsatellite data using the *poppr* package version 2.9.6 (Kamvar et al. 2014) in R version 4.4.0 (R Core Team 2022). Gene diversity for each locus was evaluated using Nei's expected heterozygosity *H*_*e*_ (Nei 1978), and PIC (Shete et al. 2000). Genotypic richness was estimated as the number of Multi‐Locus Genotypes (MLGs), inferred through the SSR allele profile, and expected Multi‐Locus Genotypes (eMLGs), obtained by performing rarefaction to the largest shared sample size among populations (Grünwald et al. 2015). MLGs were defined as isolates identical across at least 14 SSR loci, which, according to the *genotype_curve* function, corresponds to the minimum number of loci needed to discriminate individual MLGs in the present dataset. Genotypic diversity was evaluated using *H*_*e*_, Simpson's index *λ* (Simpson 1949), and its correction based on the sample size (*N*) for each subpopulation using the following formula: [*N*/(*N* − 1)] × *λ* (Grünwald et al. 2015). Prior to population structure analyses, clone correction was performed with the *clonecorrect* function to avoid over‐representation of epidemic MLGs. When multiple isolates shared the same MLG, a single representative was retained for each subpopulation based on sampling year and geographic origin. To investigate relationships between MLGs based on geographic origin and sampling year, minimum spanning networks were built using Bruvo's distance (Bruvo et al. 2004). Genetic clusters (K) based on SSR genotyping were determined using Structure version 2.3.4 on the clone corrected dataset, as the clustering algorithms employed by the software assume independent genotypes and can be biased by the presence of clones (Pritchard et al. 2000). Different *K* values (from 1 to 10) were compared in 15 independent runs for each K using 200,000 Markov Chain Monte Carlo iterations after a burn‐in period of 200,000 repetitions, assuming an admixture model with no prior information on isolates origin. The optimal number of *K* was estimated with the StructureSelector online tool (Li and Liu 2018) according to the Evanno Δ*K* statistical method (Evanno et al. 2005). Cluster assignment for each genotype was achieved through Discriminant Analysis of Principal Components (DAPC) using the R packages *adegenet* version 2.1.10 (Jombart et al. 2010) and visualized with *ade4* version 1.7‐22 (Dray and Dufour 2007). The *find.clusters* function allowed to estimate the optimal number of K based on the Bayesian Information Criterion (BIC) and the *optim.a.score* function was used to infer the optimal number of principal components to retain in the DAPC. The optimal number of *K* was estimated with the StructureSelector online tool (Li and Liu 2018) according to the Evanno Δ*K* statistical method (Evanno et al. 2005). Ultimately, an Unweighted Pair Group Method with Arithmetic mean (UPGMA) dendrogram with a 10,000‐replicate bootstrap based on Nei's distance was used to validate the optimal number of K by coloring each genotype with its DAPC cluster assignment. The amount of genetic variance explained by DAPC‐inferred clusters, as well as the influence of sampling year and location on population differentiation, was also assessed through Analysis of Molecular Variance (AMOVA) (Excoffier et al. 1992) with 999 permutations for significance testing using the *poppr* and *ade4* packages. Population divergence based on cluster analysis, sampling year, and location was investigated using pairwise Hedrick's *G*_*st*_ index (Hedrick 2005), ranging from 0, no differentiation, to 1, total divergence, with the *mmod* package version 1.3.3 (Winter 2012). To evaluate whether cluster membership was associated with sampling year or geographic origin, contingency tables of cluster × region and cluster × year were built from the isolate‐level cluster assignment data. Pearson's chi‐square test of independence was applied to each contingency table, and *p*‐values were estimated by Monte‐Carlo simulation with 10,000 replicates. For post‐hoc identification of which cells contributed to a significant table result, standardized Pearson residuals were computed as (Observed − Expected)/√Expected for each cell. *p*‐values for the residuals were corrected for multiple testing across all table cells using the Benjamini–Hochberg procedure (false discovery rate, FDR). Cells with FDR *q* < 0.05 were considered significant. Isolation‐by‐distance was tested using a Mantel test between pairwise geographic distances and pairwise genetic distances (Euclidean distance on the multilocus SSR genotype matrix). Significance of Spearman's rank correlation was assessed by 999 permutations using the *Mantel* function in the *vegan* R package (Oksanen et al. 2022).

### 2.4 Correlation Analysis of Genetic Diversity and Meteorological Variables

In addition to sampling year and location, temperature, relative humidity (RH), and rainfall were considered as possible factors affecting *P. oryzae* genetic diversity. This approach explores the hypothesis that, within a fixed location, years with climatic conditions closer to *P. oryzae*'s biological optimum (warm, humid conditions and sufficient rainfall) are expected to generate larger local epidemics, therefore increasing effective population size and the opportunity to maintain and/or generate genotypic diversity (McDonald and Linde 2002). Only isolates from Piedmont region were considered, due to the adequate sample size among different years (76 in 2011, 65 in 2012, 14 in 2020, 13 in 2021, 15 in 2022), whereas in Lombardy only 3 and 2 isolates were obtained in 2011 and 2012, respectively (Figure 1, Table S1). Genotypic diversity (*H*_*e*_ and *λ*) was calculated on the Piedmontese subpopulations for each sampling year. Monthly meteorological data were obtained from ARPA Piemonte (https://www.arpa.piemonte.it/rischi_naturali/snippets_arpa_graphs/map_meteoweb/?rete=stazione_meteorologica) from June to October, corresponding to the rice cropping season in Italy and the occurrence of blast disease symptoms. Six weather stations were considered, including two in Novara province (Novara and Cameri) and two in Vercelli province (Casello Ruggerina and Albano Vercellese), representing the most sampled areas in Piedmont, whereas only one station was considered for Biella (Salussola) and Alessandria (Casale Monferrato) provinces (Figures S1–S2). For each weather station, monthly temperature (°C) and RH (%) were averaged to the sampling year, while monthly rainfall (mm) was converted into yearly by sum. All meteorological data were averaged among weather stations for correlation analysis with genetic diversity using Pearson test (*p* < 0.05), after checking for data normality. Rainfall data were log‐transformed after observing a non‐normal data distribution, according to Shapiro–Wilk test (*p* < 0.05).

### 2.5 Virulence Assessment on Rice Leaves

Eight strains, selected as representative of the DAPC inferred subpopulations, were deposited at Turin University Culture Collection (Table S3) and tested for their virulence on susceptible rice cultivar Vialone Nano, a commonly grown, highly susceptible Italian cultivar lacking known major blast R genes (Bagnaresi et al. 2012), under controlled conditions. This exploratory trial aimed at verifying whether strains belonging to different genetic clusters may also display variable baseline virulence. Artificial inocula were prepared according to Tacconi et al. (2010) with few adjustments. Fungal cultures were grown on a substrate prepared with 20 g L^−1^ of rice flour, 2.5 g L^−1^ of yeast extract (Merck, Germany), 15 g L^−^^1^ of agar (VWR, Italy), and 0.025 g L^−1^ of streptomycin sulfate (Merck), added after sterilization, at room temperature (22 ± 2°C) for 15 days. Vialone Nano seeds were sown in a substrate made of 60% peat and 40% sand inside plastic trays by dividing 30 seeds into 3 rows. The trays were placed in a growth chamber under controlled conditions at 24 ± 1°C with a photoperiod of 12 h. Two weeks after seed germination, corresponding to 48 h before artificial inoculation, rice plants were fertilized with ammonium nitrate (8.6 grams equivalent of N in 1 L of sterile distilled water). To prepare the inoculum, *P. oryzae* conidia were collected by flooding the plates with sterile Ringer solution amended with 0.5% sodium carboxymethyl cellulose (CMC‐Na, Merck), followed by scraping the surface with a sterile loop, filtering through two layers of sterile gauze, and adjusting the concentration to 5 × 10^4^ conidia mL^−1^ with a Burker chamber. Rice plants were spray inoculated until runoff (50 mL of conidial suspension for each tray), and the trays were covered with a plastic tarp for 48 h to raise humidity and facilitate the pathogen infection. One week after inoculation, disease severity was scored using a scale with values from 1, absence of leaf blast lesions, to 5, extended sporulating lesions, according to Faivre‐Rampant et al. (2011). For each *P. oryzae* strain, 3 trays were inoculated, for a total of 24 trays. The experiment was performed twice. Disease severity (DS) scores of each individual plant were converted to percentage values for each biological replicate according to (McKinney 1923) with the following formula:

$$\mathrm{DS}\%=\frac{\sum \left(s\times F\right)}{N\times m}\times 100,$$

where: *s* = DS score (1, 2, 3, 4, 5); *F* = frequency of each DS score; *N* = number of plants for each tray, thus biological replicate; *m* = maximum value of the DS scale. Analysis of variance was performed in R environment, after assessing data normality and homoscedasticity, and Tukey's HSD test was used for pairwise comparisons between strains (*p* < 0.05).

## 3 Results

### 3.1 Genetic Diversity of *P. oryzae* in Italy

**FIGURE 2:**
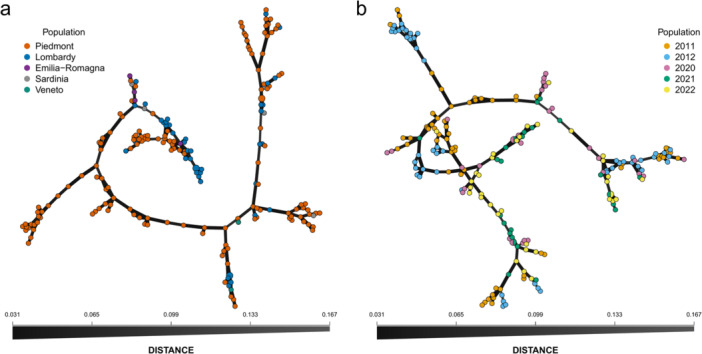
Minimum spanning networks of 200 Italian *P. oryzae* MLGs based on geographic origin (a) and sampling year (b) using Bruvo's genetic distance. Node size and color represent the number of MLGs and subpopulation, respectively.

A total of 242 monoconidial isolates were obtained, corresponding to the number of sites considered in the sampling, and molecularly identified with species‐specific primers before proceeding with genotyping. Two strains (NO5.1 and PV11.2, both isolated from samples collected in 2020, respectively from Piedmont and Lombardy) were discarded from the analysis as they showed 25% of missing data across the investigated SSR regions. Locus‐specific metrics are reported in Table 1. Marker pyrms37 was found to be monomorphic in all samples. The number of observed alleles ranged from 2 in MGM438 to 15 in MGM266, with an average of 5.75 due to most loci (5) displaying only 3 alleles. Mean gene diversity (*H*_*e*_) among loci was 0.47, with maximum values in MGM429, MGM437, and MGM266, with *H*_*e*_ values of 0.90, 0.87, and 0.86, respectively, whereas pyrms409, pyrms385, and pyrms685 displayed the lowest *H*_*e*_ values of 0.12, 0.17, and 0.22, respectively. PIC followed the same trend of *H*_*e*_, with an average of 0.47, a maximum value of 0.89 in MGM 429 and a minimum of 0.14 in pyrms409. Genotypic diversity measures of the 240 *P. oryzae* isolates based on sampling year are listed in Table 2. The highest number of MLGs was detected in 2011, followed by 2012, respectively, 61 and 51. However, after rarefaction to the lowest sample size, higher genotypic richness was detected in 2020, followed by 2022 and 2021, with, respectively, 27.1, 26.8, and 25 eMLGs. Clone correction allowed to obtain 200 unique MLGs, corresponding to a clonal fraction of 17.4%, consistent with a Pareto‐like distribution (Arnaud‐Haond et al. 2007). This dataset was used to evaluate genotypic diversity using Simpson's index (*λ*), *λ* corrected based on sample size for each sampling year, and *H*_*e*_ (Table 2). Highest *λ* values were detected in 2011, followed by 2012 (respectively, 0.984 and 0.980), whereas *λ* corrected peaks were in 2020 (0.998), 2022 (0.997), and 2021 (0.992). Nei's expected heterozygosis values, on the other hand, were not affected by sample size, as the highest *H*_*e*_ values were in 2020 (0.458), 2022 (0.450), and 2021 (0.436). Overall, genotypic diversity was high in the whole population, with average values of *λ* corrected and *H*_*e*_ of 0.998 and 0.482, respectively. Genetic relationships among MLGs were investigated both in terms of samples collection time and location through minimum spanning networks on the clone‐corrected dataset using Bruvo's genetic distance (Figure 2). No relationship between subpopulations was observed, as nodes belonging to the same sampling location or year are sparse throughout the network, suggesting the lack of a strong geographical or temporal structuring. The only MLGs shared among different subpopulations were identified between 2020 and 2021 (MLG77, in two different locations in Piedmont, and MLG100, both in Lombardy) and between 2021 and 2022 (MLG130, both in Lombardy).

**TABLE 2:** Genotypic richness of the Italian *P. oryzae* collection, estimated as the number of Multi‐Locus Genotypes (MLG) and expected number MLG (eMLG) after rarefaction to the smallest sample size (N) ± standard error (SE). Genotypic diversity of the clone‐corrected dataset, expressed as Simpson's index (*λ*) and its correction based on sample size (*N*), and Nei's expected heterozygosity (*H*_*e*_).

Population	Whole collection			Clone‐corrected			
	*N*	MLG	eMLG ± SE	*N*	*λ*	*λ* corrected	*H*_*e*_
2011	81	61	24.8 ± 1.5	61	0.984	0.990	0.424
2012	67	51	24.8 ± 1.4	51	0.980	0.991	0.432
2020	30	29	27.1 ± 0.3	29	0.966	0.998	0.458
2021	28	25	25.0 ± 0.0	25	0.960	0.992	0.436
2022	36	34	26.8 ± 0.7	34	0.971	0.997	0.450
Total	242	200	27.2 ± 0.9	200	0.995	0.998	0.482

### 3.2 Mating Type Analysis

Mating type for each isolate was determined by PCR assay, using European reference strains from Roumen et al. (1997), all belonging to Mat1.1 idiomorph, as positive controls. A no template control and *F. fujikuroi* strain A5 were used as negative control for both Mat1.1 and Mat1.2. All isolates tested positive for Mat1.1 by PCR, and no amplification was observed using the Mat1.2 primer pair. Idiomorph identity was confirmed by Sanger sequencing of 50 representative strains, 10 for each sampling year, and comparison with Mat1.1 sequences deposited in Genbank public database (National Institutes of Health, NIH). These results confirm the presence of only Mat1.1 idiomorph in Italy and exclude possible sexual reproduction in the local *P. oryzae* population.

### 3.3 Cluster Analysis of Italian *P. oryzae* Population

**FIGURE 3:**
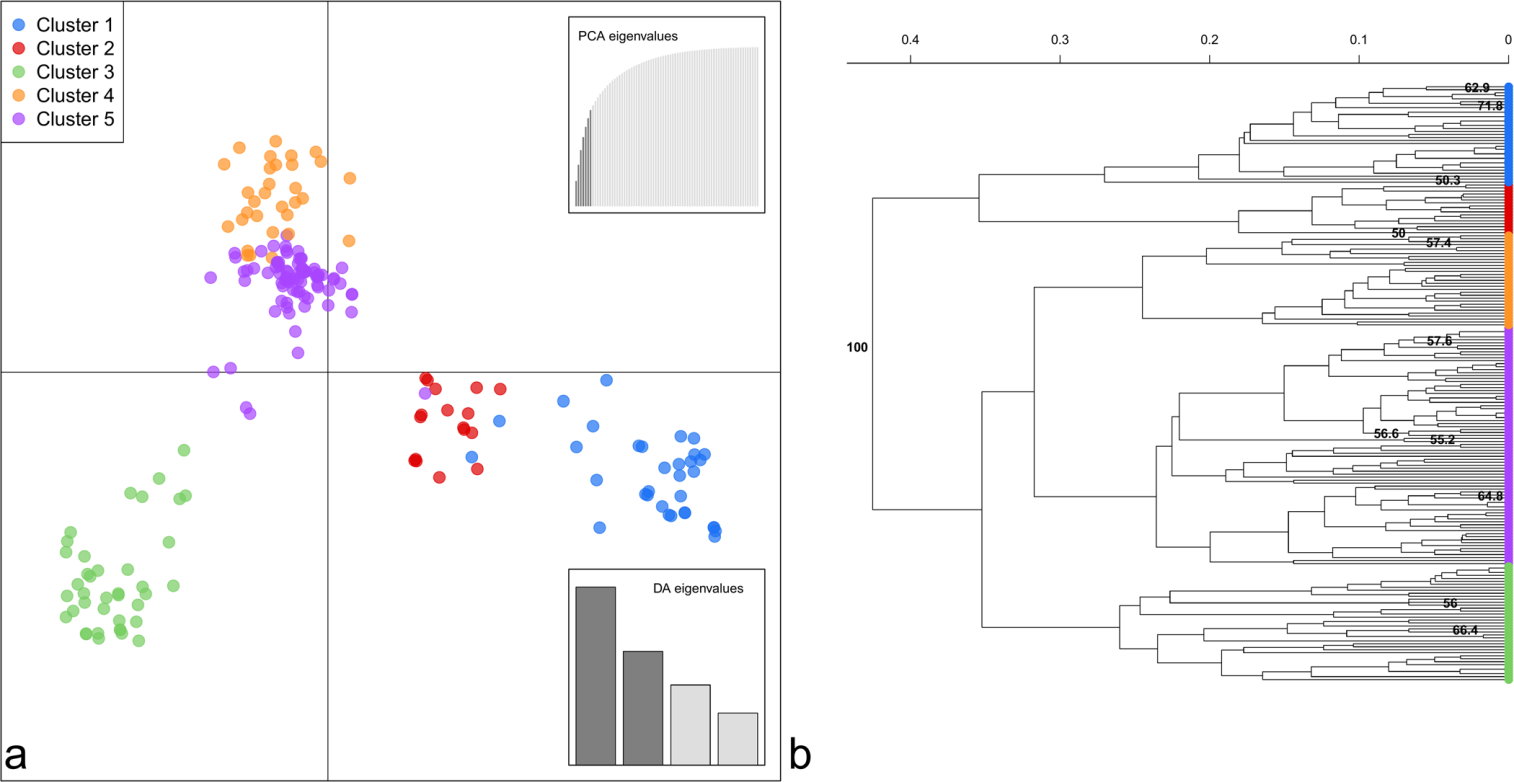
Population structure of 200 Italian *P. oryzae* isolates assigned to five genetic clusters based on DAPC analysis (a) and UPGMA dendrogram with a 10,000‐replicate bootstrap using Nei's genetic distance (b). Terminal nodes of the dendrogram are colored based on DAPC cluster assignments (b).

The population structure of the clone‐corrected *P. oryzae* dataset was determined using DAPC, Structure, and UPGMA dendrogram clustering analyses. The optimal number of *K* with the DAPC method was estimated by observing an “elbow” at *K* = 5 in the curve of BIC values as a function of the number of *K* (Figure S3a). The output from the Structure analysis was uploaded in the StructureSelector online tool, which, following the Evanno Δ*K* statistical method, allowed to detect a peak Δ*K* value corresponding to an optimal number of *K* = 5 (Figure S3b). Cluster assignments for each genotype were obtained with the *find cluster* function and visualized through a DAPC scatterplot (Figure 3a, Table S4). As clusters 4 (orange) and 5 (purple) were partially overlapping, projection on axes 2 and 3 of the DAPC plot allowed to observe a clearer separation between these genetic groups (Figure S4). Additionally, an UPGMA dendrogram based on Nei's genetic distance with a 10,000‐replicate bootstrap was built, and the DAPC cluster assignments were plotted on each terminal node to further confirm the inferred subpopulations (Figure 3b). An upper hierarchical subdivision can be noticed in the dendrogram, where clusters 1 (blue) and 2 (red) group together, clearly separated from clusters 3 (green), 4 (orange), and 5 (purple), with a bootstrap value of 100 (Figure 3b). Genotypic diversity was calculated for each DAPC‐inferred subpopulation as illustrated in Table 3. Cluster 5 included the highest number of isolates (80), whereas cluster 1 only presented 17 individuals. Simpson's diversity index ranged from 0.998 (cluster 2) to 1 (clusters 1 and 4) after sample size correction, whereas Nei's expected heterozygosis allowed to better highlight differences among subpopulations, with the highest *H*_*e*_ value detected in cluster 3 (0.351), followed by clusters 5 (0.321) and 4 (0.293), whereas clusters 2 (0.264) and 1 (0.180) displayed lower allelic diversity. A second DAPC analysis including representative strains of European *P. oryzae* population (Roumen et al. 1997; Thierry et al. 2022) allowed to investigate the presence of new genetic groups arising from recent *P. oryzae* Italian populations. Cluster assignments were plotted on a UPGMA dendrogram based on Nei's genetic distance with a 10,000‐replicate bootstrap (Figure S5). Strain IT0003, representative of European lineage 5 (Roumen et al. 1997), and strain PR0025 (Thierry et al. 2022) grouped in cluster 1; IT0010, FR0013 and SP0001, representative of lineage 1, grouped in cluster 2; IT0002, representative of lineage 2, as well as strains IT0046, FR0160 and PR0009, grouped in cluster 3; HN0001 and SP0006, representative of, respectively, lineages 3 and 4, as well as strains IT0104 and HN0006, grouped in cluster 5, indicating that cluster 4 is unique to the Italian *P. oryzae* population considered in this study.

**TABLE 3:** Genotypic diversity of the DAPC‐inferred clone‐corrected Italian *P. oryzae* genetic subpopulations, estimated as the number of Multi‐Locus Genotypes (MLG), Simpson's index (*λ*) and its correction based on sample size (*N*), and Nei's expected heterozygosity (*H*_*e*_).

Cluster	*N*	MLG	*λ*	*λ* corrected	*H*_*e*_
1	17	17	0.941	1	0.180
2	33	32	0.968	0.998	0.264
3	39	38	0.973	0.999	0.351
4	31	31	0.968	1	0.293
5	80	79	0.987	0.999	0.321
Total	200	197	0.995	0.999	0.482

### 3.4 Effect of Year and Location on Genetic Diversity

**FIGURE 4:**
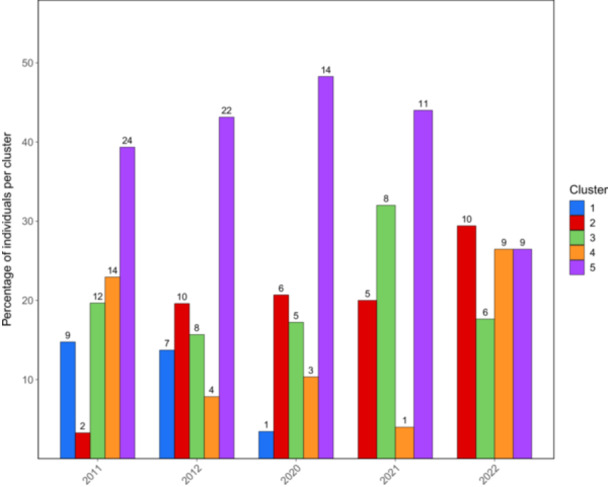
Distribution of 200 Italian *P. oryzae* strains based on their DAPC‐inferred genetic clusters for each year of sample collection. Data are presented both as absolute frequencies, indicated on top of each bar, and as a percentage of strains belonging to a genetic cluster on the *y*‐axis.

AMOVA was performed to assess the main factors driving population differentiation (Table 4). The percentage of variance explained by factors related to the isolates origin was significant (*p* = 0.001) among subpopulations based on sampling year (14.5%) and region (9.7%). However, the highest significant variation was among DAPC‐inferred clusters (49%). Cluster composition among different sampling years and regions was also investigated as a first clue of a possible effect of these factors on genetic diversity (Table S5). Additionally, genetic divergence among subpopulations based on sampling year and location, as well as DAPC cluster assignment, was estimated using pairwise Hedrick's *G*_*st*_ index (Table 5). Genetic differentiation was higher among DAPC‐inferred clusters, with *G*_*st*_ values ranging from 0.496 (between clusters 3 and 5) to 0.826 (between clusters 1 and 4) and a mean overall value of 0.70, compared to 0.21 and 0.23 based on, respectively, sampling year and region. Divergence among subpopulations based on sampling region ranged from 0.186 to 0.342, whereas the *G*_*st*_ value between the most sampled regions (Piedmont and Lombardy) corresponded to 0.227. Contingency analysis indicated a significant association between genetic cluster and sampling region (Pearson's *χ*², Monte‐Carlo *P* = 3.0 × 10^−4^; *X*² = 44.785). Post‐hoc analysis of standardized Pearson residuals revealed that cluster 2 was over‐represented in Lombardy (*P* ≈ 1.27 × 10^−6^; *q* = 3.17 × 10^−5^); however, no other region × cluster cells remained significant after multiple test correction. A Mantel test (Spearman, 999 permutations) between geographic distance and genetic distance returned a small but significant correlation (Mantel *r* = 0.132, *p* = 0.001), indicating weak isolation by distance across sampled sites. Based on geographic origin, all five clusters were found in the most sampled regions (Piedmont and Lombardy) in similar proportions. However, cluster 2 was the most represented in Lombardy, comprising 46.5% of the isolates, while cluster 5 was predominant in Piedmont, accounting for 43.8%. Concerning cluster composition changes over time, all DAPC‐inferred genetic groups were detected in early sampling years, with the exception of cluster 1, which showed a decreasing presence, with 14.8% individuals assigned to this subpopulation in 2011, 13.7% in 2012 and 3.5% in 2020, until not being detected in 2021 and 2022 (Figure 4). On the other hand, cluster 5 was the most represented in almost all sampling years, with 39.3% isolates in 2011, 43.1% in 2012, 48.3% in 2020, and 44% in 2021. In 2022, cluster 2 was the main group detected (29.4%). The highest *G*_*st*_ value detected among different years was between 2021 and 2011 (0.338) and, in general, between the sampling campaigns 2011–2012 and 2020–2022, whereas 2011 and 2012 (*G*_*st*_ = 0.140), as well as subpopulations sampled in 2020–2022 (*G*_*st*_ ranging from 0.057 to 0.063), showed less genetic differentiation. The association between genetic cluster and sampling year was also significant at the contingency table level (Pearson's *χ*², Monte‐Carlo *p* = 0.0037; *X*² = 35.21). However, after FDR correction of cellwise residual *p*‐values, no individual year × cluster cell remained significant, thus the year effect cannot be attributed to a single, clearly identified year × cluster enrichment.

**TABLE 4:** Analysis of molecular variance (AMOVA) of the clone‐corrected Italian *P. oryzae* collection for each hierarchical level of sampling year and region, as well as DAPC‐inferred genetic subpopulations. Significance testing was performed with 999 permutations.

AMOVA	Df	Variance (σ)	Variance (%)	*p* value
Between samples within years	4	0.81	14.49	
Within samples	195	4.77	85.51	
Total	199	5.58	100	0.001
Between samples within regions	4	0.56	9.71	
Within samples	195	5.16	90.29	
Total	199	5.72	100	0.001
Between samples within clusters	4	3.02	49.06	
Within samples	195	3.14	50.94	
Total	199	6.16	100	0.001

**TABLE 5:** Genetic divergence of the clone‐corrected Italian *P. oryzae* collection based on sampling year and region, as well as DAPC‐inferred genetic subpopulations, based on Pairwise Hedrick's *G*_*st*_.

Pairwise Hedrick's *G*_*st*_				
Year	2011	2012	2020	2021
2012	0.140			
2020	0.259	0.249		
2021	0.338	0.314	0.057	
2022	0.292	0.270	0.063	0.057
Region	Piedmont	Lombardy	Veneto	Emilia‐Romagna
Lombardy	0.227			
Veneto	0.196	0.186		
Emilia‐Romagna	0.216	0.285	0.186	
Sardinia	0.270	0.342	0.217	0.276
Cluster	5	1	3	4
1	0.752			
3	0.496	0.805		
4	0.523	0.826	0.593	
2	0.656	0.754	0.743	0.736

**FIGURE 5:**
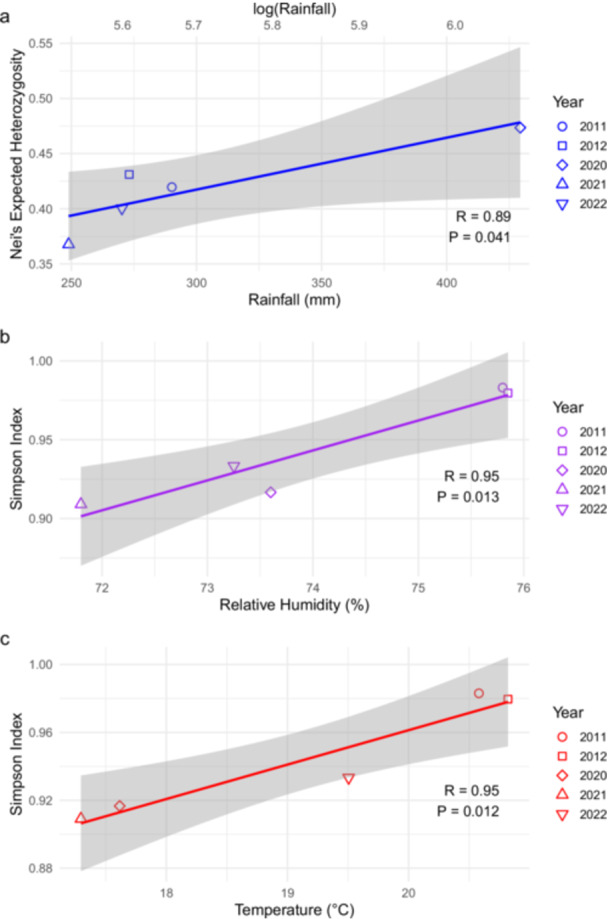
Correlation analysis between genotypic diversity (*H*_*e*_ and *λ*) of *P. oryzae* isolates from Piedmont and meteorological variables, that is, rainfall (a), relative humidity (b), and temperature (c), obtained from weather stations located in Piedmont for each sampling year.

Despite geographical origin explained a limited amount of the observed genetic differentiation, additional variables related to location, that is, rainfall, RH, and temperature, were collected from Piedmontese weather stations for each sampling year and used for a correlation analysis with genotypic diversity measures (*H*_*e*_ and *λ*) of Piedmontese isolates. Results are illustrated in Figure 5, where it can be observed that, in this case, the highest diversity in terms of *H*_*e*_ was found in Piedmontese isolates from, in descending order, 2020, 2012, 2011, 2022, and 2021, whereas the highest *λ* was detected in 2011, followed by 2012, 2022, 2020, and 2021. Rainfall significantly correlated with Nei's expected heterozygosis (*p* = 0.04), whereas RH and temperature were more significantly correlated with Simpson's index (*p* = 0.01). All meteorological variables showed a strong positive correlation with genotypic diversity, with Pearson's correlation coefficient (*R*) ranging from 0.89, for rainfall with *H*_*e*_, to 0.95, for RH and temperature with *λ*. These results indicate that years with climatic conditions closer to the pathogen's reported optima (Katsantonis et al. 2017) tended to show higher measured genotypic diversity in our sample.

### 3.5 Virulence of Representative Strains

Eight *P. oryzae* strains, representative of the DAPC‐inferred genetic clusters (PV4.2 for cluster 1; MI4 and NO4.2 for cluster 2; CR1 and LOM4b for cluster 3; LOM5 for cluster 4; LOM8 and VC2.3 for cluster 5), were inoculated on susceptible rice cultivar Vialone Nano to assess their virulence (Figure S6). This preliminary test was conducted following the hypothesis that more virulent strains can outcompete and lead to the gradual disappearance of less aggressive subpopulations, as it was observed with cluster 1 in the present work. Analysis of variance of disease severity (DS) data, followed by Tukey's HSD test, allowed to detect significant differences in virulence among strains. The highest DS% value was determined by CR1 (56.2%), followed by VC2.3 (55.3%) and LOM4b (54.7%), which were significantly more virulent than strain MI4, causing an average DS% of 35.1%. In general, strains belonging to clusters 1 (PV4) and 2 (MI4 and NO4.2) were less virulent, whereas the strain of cluster 4 (LOM5) showed an intermediate average value of DS% (45.3%). Strains belonging to cluster 5 were variable in virulence levels, with only VC2.3 having a significant effect compared to MI4, whereas LOM8 showed an intermediate virulence level (48%). Both strains of cluster 3 (CR1 and LOM4b) determined significantly higher DS% compared to MI4.

## 4 Discussion

To explore the genetic variability of the local *P. oryzae* population, the main Italian rice cultivation areas (Piedmont, Lombardy, Emilia‐Romagna, Veneto, and Sardinia) were sampled during two campaigns, 2011–2012 and 2020–2022, and isolates were genotyped using SSR markers. A total of 242 strains were genotyped using 16 SSR markers previously developed for population studies. High genotypic richness and a low clonal fraction were detected, especially in the more recent sampling campaign (2020–2022). The clone‐corrected dataset also showed elevated genotypic diversity, with average Simpson's index and Nei's expected heterozygosity values of, respectively, 0.998 and 0.482, similar to those reported in other *P. oryzae* population studies using SSR markers (D'Ávila et al. 2016; Le et al. 2023; X. Wang et al. 2017). Overall, the highest genetic diversity was found in 2020, followed by 2022 and 2021. PCR amplification of mating type locus confirmed the clonal nature of the Italian *P. oryzae* population. All the isolates displayed the Mat1.1 idiomorph, in agreement with previous studies stating that Mat1.2 was never reported in Europe (Lara‐Álvarez et al. 2010; Nottéghem and Silue 1992).

Genetic relationships among MLGs through minimum spanning networks based on sampling year and geographical origin showed no clear grouping patterns among subpopulations. However, cluster analysis on clone‐corrected dataset, combining different approaches to infer the most probable number of K without *a priori* information on the origin of the isolates, showed the presence of five groups. Reference strains of *P. oryzae* genetic diversity in the EU from Roumen et al. (1997) and Thierry et al. (2022) were assigned to clusters 1, 2, 3, and 5, indicating that one of the inferred genetic clusters in our study was not sampled in these previous studies. The main factors driving population differentiation were explored using AMOVA and Hedrick's *G*_*st*_ index. In both cases, a higher degree of genetic divergence among subpopulations was explained by sampling year rather than geographical origin. The influence of time and geographic origin on *P. oryzae* Italian population was also explored by assessing the cluster distribution according to sampling year and location. Cluster 2 was the most represented in Lombardy and cluster 5 in Piedmont. On the other hand, cluster 1 gradually disappeared from the *P. oryzae* subpopulations based on sampling year, being well represented in 2011–2012, including few isolates in 2020 and then not being detected in 2021 and 2022, highlighting the dynamic genetic changes in the Italian population over time. The chi‐square analyses showed that cluster composition is not independent of sampling region and year, indicating a certain degree of spatial and temporal structuring of the Italian *P. oryzae* population. The spatial association was driven primarily by the significant over‐representation of cluster 2 in Lombardy, whereas temporal structure was detectable at the contingency table level but not attributable to any single year after correction for multiple testing. Additionally, although the Mantel test shows a significant positive relationship between geographic and genetic distances, the effect size is small (*r* = 0.132), suggesting geographic distance explains little of the genetic variation. It has often been reported, however, that the rice blast pathogen displays high genetic diversity irrespective of country of origin and ability to reproduce sexually. Wang et al. (2017) genotyped 427 isolates collected in seven states from 1970s to 2010s in the United States, reporting greater genetic diversity among sampling campaigns than among geographical areas, consistent with the findings of our study. Yadav et al. (2019) and Odjo et al. (2021) also found that location was not the primary factor driving *P. oryzae* genetic differentiation in India and Africa, respectively, suggesting the exchange of infected seeds as a major driver of gene flow into local population dynamics. Concerning Europe, Lara‐Álvarez et al. (2010) also suggested the role of seeds as the primary source of inoculum being the most important factor affecting *P. oryzae* diversity in Spain, with the presence of a local distinct lineage compared to those inferred by Roumen et al. (1997). The stochastic introduction of infected seed as a source of *P. oryzae* genetic variability, however, may not be the most important factor in this case, as seed production of most rice varieties cultivated in Italy takes place within the country.

In Italy, Piotti et al. (2005) did not observe such genetic divergence from reference strains of European *P. oryzae* diversity detected by Roumen et al. (1997), with the exception of two unique haplotypes that did not cluster with any of the lineages they defined. In their work, reference strain IT0003 grouped in Lineage 1, PR3 and PR72, which clustered in the same lineage of IT0002 in Roumen et al. (1997), grouped in Lineage 2, FR0013 grouped in Lineage 3, whereas HN0001 and SP0006 did not cluster with any of the lineages defined based on rep‐PCR analysis on *Pot2* region. Our studies cannot be directly compared, mainly due to the differences in molecular markers type and number used for the population structure analysis, which led to observe different distances among the genetic groups. In Piotti et al. (2005), Lineage 1, which could be associated to our cluster 1, is genetically closer to Lineage 2, which may correspond to our cluster 3, whereas Lineage 3, which could be associated to our cluster 2, is distinct from the previous lineages. Strains HN0001 and SP0006 were assigned to cluster 5 through DAPC analysis in our study, however in the UPGMA dendrogram (Figure S5) it can be observed how they are also clearly separated from members of the same cluster, which is aligned with the results from Piotti et al. (2005). The observed genetic distance from cluster 5 of representative strains HN0001 and SP0006 from Roumen et al. (1997) in the UPGMA dendrogram may indicate that cluster 5 was not sampled by Roumen et al. (1997). Additionally, strain IT104 from Thierry et al. (2022) grouped within cluster 5, supporting the presence of Italian genotypes not represented in Roumen et al. (1997) collection. The present study, therefore, reports the presence of one cluster including only isolates from the present study, as representative strains from Thierry et al. (2022) did not cover the whole genetic diversity in the Italian population collected in this work. We cannot exclude, however, that the unique haplotypes found in Piotti et al. (2005) could also belong to cluster 4, or that the addition of molecular markers to analyze their strains could lead to more comparable results.

To investigate the combined effect of sampling year and geographical origin, meteorological variables from the most evenly sampled region through time (Piedmont) were correlated with the genotypic diversity of the pathogen. Temperature and relative humidity (RH), as well as rainfall to a lesser extent, showed a statistically significant and strong positive correlation with *P. oryzae* allelic diversity in a selected set of isolates from Piedmont. It has been previously suggested that high genotypic diversity in clonally reproducing populations may be linked to the successful adaptation to different environmental niches (Kassen and Rainey 2004; Thierry et al. 2022), whereas lower genetic diversity would imply the presence of a bottleneck process (Sillo 2022), e.g., the selection of few fungicide‐resistant strains (D'Ávila et al. 2021). Here, we also hypothesized that years characterized by favorable environmental conditions within a fixed location (temperature and relative humidity near the pathogen's optimum and sufficient rainfall) produce larger pathogen epidemics by increasing the number of asexual reproduction cycles per season. Larger population sizes reduce the strength of genetic drift and increase the pool of genetic variation and the rate at which new variants arise (McDonald and Linde 2002), therefore, expecting to measure higher allelic diversity in samples collected in those years. The positive correlation between *P. oryzae* genetic diversity and meteorological variables in Piedmont is in agreement with this theory: the average yearly temperature and RH collected from the weather stations were around 21°C and 76% for the sampling years displaying the highest allelic diversity, and it is well established that *P. oryzae* optimal temperature and humidity conditions during its life cycle range from 20°C to 28°C and 89% to 96%, respectively (Katsantonis et al. 2017). A causal relationship between meteorological variables and genetic diversity, however, should be further corroborated through specific experimental designs aimed at multi‐year sampling and genotyping of *P. oryzae* with a higher number of fixed locations next to weather stations. Taken together, our results did not allow to find the main determinants of the observed high allelic diversity in the Italian *P. oryzae* population, despite the presence of only one mating type. Park et al. (2008) and D'Ávila et al. (2016) found high genetic differentiation between *P. oryzae* isolates based on the rice cultivar of origin. Future efforts, therefore, should focus on further confirming the link between pathogen and host genetic diversity, as well as to investigate the influence of cultivation methods, that is, flooded or dry systems (Mongiano et al. 2021), as a possible determinant of *P. oryzae* allelic diversity.

Reference strains from the DAPC‐inferred clusters were chosen to investigate the possible role of virulence on rice blast susceptible cultivar Vialone Nano as a factor explaining the gradual disappearance of cluster 1 through time. No significant differences were detected among clusters, except for two representative strains of cluster 3, which were both more virulent than one of the two representative strains of cluster 2. In general, however, a trend was observed, where clusters 1 and 2 displayed a generally lower virulence compared to the other three clusters, which are also genetically close according to the DAPC scatterplot and UPGMA dendrogram. These results could represent one of the possible explanations of the gradual disappearance of cluster 1, probably being replaced by more aggressive subpopulations. Virulence was assessed with a disease severity scale evaluating the presence of necrotic lesions showing distinct sporulation and their size (Faivre‐Rampant et al. 2011). For foliar pathogens such as *P. oryzae*, larger lesions commonly yield more conidia (Maina and Oerke 2024), theoretically allowing more aggressive strains to be more ecologically competitive. The relatively low overall proportion of isolates belonging to the UPGMA clade, including clusters 1 and 2 (50), compared to the clade comprising the remaining genetic groups (150), could reinforce this hypothesis. Further trials on a wider set of Italian commercial susceptible rice cultivars, however, are needed to prove this theory, as well as testing a higher number of representative strains of Italian *P. oryzae* diversity. Additionally, whole genomes of reference strains will be sequenced to investigate the presence of cluster‐specific effectors.

## 5 Conclusions

In conclusion, this work aimed at defining the spatial, temporal, environmental, and biological determinants of *P. oryzae* genetic diversity in Italy through SSR genotyping. The overall population structure was maintained over time, as no new genetic clusters emerged in a decade, and includes unique genetic diversity that hasn't been detected so far in reference strains of European diversity. We theorize that the high observed allelic diversity might have been affected by environmental factors, including temperature, RH, and rainfall. Additionally, we explored the role of virulence in the disappearance of a genetic cluster over time, which will require further investigation. This work set the basis for the strategic deployment of known R genes in Italian breeding programs against the AVR effectors that are actually present in the local *P. oryzae* population, which will be detected through whole‐genome sequencing of reference strains identified in this study.

## Author Contributions

**Simone Bosco:** conceptualization (supporting), formal analysis (lead), writing – original draft (lead), writing – review and editing (equal), **Fabiano Sillo:** conceptualization (supporting), formal analysis (supporting), writing – original draft (supporting), writing – review and editing (equal). **Paola Ruffa:** formal analysis (supporting), writing – review and editing (equal). **Lisa Bergonzi:** formal analysis (supporting). **Daniele Tenni:** formal analysis (supporting). writing – review and editing (equal). **Pamela Abbruscato:** formal analysis (supporting). **Daniela Torello Marinoni:** conceptualization (supporting), formal analysis (supporting), writing – review and editing (equal). **Marco Romani:** formal analysis (supporting). **Davide Spadaro:** conceptualization (lead), formal analysis (supporting), writing – review and editing (equal).

## Conflicts of Interest

The authors declare no conflicts of interest.

## Supporting information


**Figure S1:** Location of Piedmontese weather stations considered for the correlation analysis. The map was made with QGIS Prizren v. 3.34.14. **Figure S2:** Monthly average values of rainfall (mm), relative humidity (%) and temperature (°C), collected from six Piedmontese weather stations, for each *P. oryzae* sampling year. **Figure S3:** Estimation of the optimal number of genetic clusters by evaluating the BIC curve as a function of the number of *K* in the DAPC analysis (a) and through the Evanno Δ*K* statistical method (b). Both analyses led to choose 5 as the optimal number of *K* due to the “elbow” in the BIC curve corresponding to this value, as well as by observing a peak in the ΔK curve at *K* = 5. **Figure S4:** Projection of the DAPC analysis of the Italian *P. oryzae* population on axes 2 and 3. **Figure S5:** UPGMA dendrogram based on Nei's genetic distance illustrating the cluster assignments of representative strains of European *P. oryzae* diversity from Roumen et al. (1997) and Thierry et al. (2022), indicated by arrows coloured based on DAPC cluster assignments, within the Italian *P. oryzae* population sampled in the present work. **Figure S6:** Virulence of eight *P. oryzae* strains, representative of Italian genetic diversity, evaluated as disease severity on leaves of 3 weeks old rice plants cultivar Vialone Nano Disease severity was scored using a scale with values from 1, absence of leaf blast lesions, to 5, extended sporulating lesions, according to Faivre‐Rampant et al. (2011). The obtained scores were subsequently converted into percentage values as described in McKinney (1923). **Table S1:** Number of *P. oryzae* isolates obtained from each province for each sampling year. **Table S2:** *P. oryzae* isolates geographic origin and sampling year. **Table S3:** Selected *P. oryzae* strains representative of the pathogen's genetic diversity in Italy. **Table S4:** *P. oryzae* strains geographic origin, sampling year and cluster assignment. **Table S5:** Absolute frequence of DAPC‐inferred clusters based on sampling year and geographic origin. Total number of isolates assigned to each cluster for each region, as well as total number of isolates from each province for each sampling year, is also reported.

## Data Availability

Datasets and R code used for the analysis and figure generation are available in the Zenodo repository at https://doi.org/10.5281/zenodo.17056136.
