# Supplementary material for: Climatic Variables and Virulence May Contribute to the Population Dynamics of Pyricularia oryzae at Local Scale
Source: Microbiologyopen. 2026 Jan 14;15(1):e70196. doi: 10.1002/mbo3.70196 (PMC12805224; doi:10.1002/mbo3.70196)
Supplement: Supplementary file 1 — Figure S1: Location of Piedmontese weather stations considered for the correlation analysis. The map was made with QGIS Prizren v. 3.34.14. Figure S2: Monthly average values of rainfall (mm), relative humidity (%) and temperature (°C), collected from six Piedmontese weather stations, for each P. oryzae sampling year. Figure S3: Estimation of the optimal number of genetic clusters by evaluating the BIC curve as a function of the number of K in the DAPC analysis (a) and through the Evanno ΔK statistical method (b). Both analyses led to choose 5 as the optimal number of K due to the “elbow” in the BIC curve corresponding to this value, as well as by observing a peak in the ΔK curve at K = 5. Figure S4: Projection of the DAPC analysis of the Italian P. oryzae population on axes 2 and 3. Figure S5: UPGMA dendrogram based on Nei's genetic distance illustrating the cluster assignments of representative strains of European P. oryzae diversity from Roumen et al. (1997) and Thierry et al. (2022), indicated by arrows coloured based on DAPC cluster assignments, within the Italian P. oryzae population sampled in the present work. Figure S6: Virulence of eight P. oryzae strains, representative of Italian genetic diversity, evaluated as disease severity on leaves of 3 weeks old rice plants cultivar Vialone Nano Disease severity was scored using a scale with values from 1, absence of leaf blast lesions, to 5, extended sporulating lesions, according to Faivre‐Rampant et al. (2011). The obtained scores were subsequently converted into percentage values as described in McKinney (1923). Table S1: Number of P. oryzae isolates obtained from each province for each sampling year. Table S2: P. oryzae isolates geographic origin and sampling year. Table S3: Selected P. oryzae strains representative of the pathogen's genetic diversity in Italy. Table S4: P. oryzae strains geographic origin, sampling year and cluster assignment. Table S5: Absolute frequence of DAPC‐inferred clusters based on sa [file MBO3-15-e70196-s001.docx]

**Table S1.** Number of *P. oryzae* isolates obtained from each province for each sampling year.

| Year | Number of isolates | Region | Province |
| --- | --- | --- | --- |
|  |  |  |  |
| 2011 | 66 | Piedmont | Novara |
| 2011 | 8 | Piedmont | Vercelli |
| 2011 | 2 | Lombardy | Pavia |
| 2011 | 2 | Piedmont | Alessandria |
| 2011 | 1 | Lombardy | Milano |
|  |  |  |  |
| 2012 | 36 | Piedmont | Vercelli |
| 2012 | 25 | Piedmont | Novara |
| 2012 | 4 | Piedmont | Alessandria |
| 2012 | 1 | Lombardy | Milano |
| 2012 | 1 | Lombardy | Pavia |
|  |  |  |  |
| 2020 | 12 | Lombardy | Pavia |
| 2020 | 7 | Piedmont | Novara |
| 2020 | 7 | Piedmont | Vercelli |
| 2020 | 3 | Emilia-Romagna | Ferrara |
| 2020 | 2 | Veneto | Verona |
| 2020 | 1 | Lombardy | Mantova |
|  |  |  |  |
| 2021 | 10 | Lombardy | Pavia |
| 2021 | 7 | Piedmont | Vercelli |
| 2021 | 4 | Piedmont | Novara |
| 2021 | 2 | Piedmont | Biella |
| 2021 | 1 | Emilia-Romagna | Ferrara |
| 2021 | 1 | Lombardy | Lodi |
| 2021 | 1 | Lombardy | Milano |
| 2021 | 1 | Sardinia | Oristano |
| 2021 | 1 | Veneto | Verona |
|  |  |  |  |
| 2022 | 12 | Lombardy | Pavia |
| 2022 | 7 | Piedmont | Novara |
| 2022 | 6 | Piedmont | Vercelli |
| 2022 | 3 | Lombardy | Milano |
| 2022 | 2 | Lombardy | Mantova |
| 2022 | 1 | Emilia-Romagna | Ferrara |
| 2022 | 1 | Lombardy | Lodi |
| 2022 | 1 | Sardinia | Oristano |
| 2022 | 1 | Veneto | Verona |
| 2022 | 1 | Piedmont | Alessandria |
| 2022 | 1 | Piedmont | Biella |
|  |  |  |  |

**Table S2.** *P. oryzae* isolates geographic origin and sampling year.

| Strain | Location | Province | Region | Year of collection |
| --- | --- | --- | --- | --- |
| ITC01 | Trino | VC | Piedmont | 2011 |
| ITC02 | Trino | VC | Piedmont | 2011 |
| ITC03 | Vigevano | PV | Lombardy | 2011 |
| ITC04 | Borgolavezzaro | NO | Piedmont | 2011 |
| ITC05 | Borgolavezzaro | NO | Piedmont | 2011 |
| ITC06 | Casalino | NO | Piedmont | 2011 |
| ITC07 | Borgolavezzaro | NO | Piedmont | 2011 |
| ITC08 | Borgolavezzaro | NO | Piedmont | 2011 |
| ITC09 | Borgolavezzaro | NO | Piedmont | 2011 |
| ITC10 | Borgolavezzaro | NO | Piedmont | 2011 |
| ITC11 | Borgolavezzaro | NO | Piedmont | 2011 |
| ITC12 | Borgolavezzaro | NO | Piedmont | 2011 |
| ITC13 | Borgolavezzaro | NO | Piedmont | 2011 |
| ITC14 | Borgolavezzaro | NO | Piedmont | 2011 |
| ITC15 | Borgolavezzaro | NO | Piedmont | 2011 |
| ITC16 | Borgolavezzaro | NO | Piedmont | 2011 |
| ITC17 | Borgolavezzaro | NO | Piedmont | 2011 |
| ITC18 | Borgolavezzaro | NO | Piedmont | 2011 |
| ITC19 | Borgolavezzaro | NO | Piedmont | 2011 |
| ITC20 | Borgolavezzaro | NO | Piedmont | 2011 |
| ITC21 | Borgolavezzaro | NO | Piedmont | 2011 |
| ITC22 | Borgolavezzaro | NO | Piedmont | 2011 |
| ITC23 | Borgolavezzaro | NO | Piedmont | 2011 |
| ITC24 | Borgolavezzaro | NO | Piedmont | 2011 |
| ITC25 | Borgolavezzaro | NO | Piedmont | 2011 |
| ITC26 | Borgolavezzaro | NO | Piedmont | 2011 |
| ITC27 | Borgolavezzaro | NO | Piedmont | 2011 |
| ITC28 | Borgolavezzaro | NO | Piedmont | 2011 |
| ITC29 | Borgo Vercelli | VC | Piedmont | 2011 |
| ITC30 | Arborio | VC | Piedmont | 2011 |
| ITC31 | Morano Po | AL | Piedmont | 2011 |
| ITC32 | Borgolavezzaro | NO | Piedmont | 2011 |
| ITC33 | Noviglio | MI | Lombardy | 2011 |
| ITC34 | Castellazzo N.se | NO | Piedmont | 2011 |
| ITC35 | Vigevano | PV | Lombardy | 2011 |
| ITC36 | Morano Po | AL | Piedmont | 2011 |
| ITC37 | S. Giacomo V.se | VC | Piedmont | 2011 |
| ITC38 | Rovasenda | VC | Piedmont | 2011 |
| ITC39 | Borgolavezzaro | NO | Piedmont | 2011 |
| ITC40 | Borgolavezzaro | NO | Piedmont | 2011 |
| ITC41 | Borgolavezzaro | NO | Piedmont | 2011 |
| ITC42 | Rovasenda | VC | Piedmont | 2011 |
| ITC43 | Borgolavezzaro | NO | Piedmont | 2011 |
| ITC44 | Casalbeltrame | NO | Piedmont | 2011 |
| ITC45 | Lignana | VC | Piedmont | 2011 |
| ITC46 | Borgolavezzaro | NO | Piedmont | 2011 |
| ITC47 | Borgolavezzaro | NO | Piedmont | 2011 |
| ITC48 | Borgolavezzaro | NO | Piedmont | 2011 |
| ITC49 | Borgolavezzaro | NO | Piedmont | 2011 |
| ITC50 | Borgolavezzaro | NO | Piedmont | 2011 |
| ITC51 | Borgolavezzaro | NO | Piedmont | 2011 |
| ITC52 | Borgolavezzaro | NO | Piedmont | 2011 |
| ITC53 | Borgolavezzaro | NO | Piedmont | 2011 |
| ITC54 | Borgolavezzaro | NO | Piedmont | 2011 |
| ITC55 | Borgolavezzaro | NO | Piedmont | 2011 |
| ITC56 | Borgolavezzaro | NO | Piedmont | 2011 |
| ITC57 | Borgolavezzaro | NO | Piedmont | 2011 |
| ITC58 | Borgolavezzaro | NO | Piedmont | 2011 |
| ITC59 | Borgolavezzaro | NO | Piedmont | 2011 |
| ITC60 | Borgolavezzaro | NO | Piedmont | 2011 |
| ITC61 | Borgolavezzaro | NO | Piedmont | 2011 |
| ITC62 | Borgolavezzaro | NO | Piedmont | 2011 |
| ITC63 | Borgolavezzaro | NO | Piedmont | 2011 |
| ITC64 | Borgolavezzaro | NO | Piedmont | 2011 |
| ITC65 | Borgolavezzaro | NO | Piedmont | 2011 |
| ITC66 | Borgolavezzaro | NO | Piedmont | 2011 |
| ITC67 | Borgolavezzaro | NO | Piedmont | 2011 |
| ITC68 | Borgolavezzaro | NO | Piedmont | 2011 |
| ITC69 | Borgolavezzaro | NO | Piedmont | 2011 |
| ITC70 | Borgolavezzaro | NO | Piedmont | 2011 |
| ITC71 | Borgolavezzaro | NO | Piedmont | 2011 |
| ITC72 | Borgolavezzaro | NO | Piedmont | 2011 |
| ITC74 | Borgolavezzaro | NO | Piedmont | 2011 |
| ITC75 | Borgolavezzaro | NO | Piedmont | 2011 |
| ITC76 | Borgolavezzaro | NO | Piedmont | 2011 |
| ITC77 | Borgolavezzaro | NO | Piedmont | 2011 |
| ITC78 | Borgolavezzaro | NO | Piedmont | 2011 |
| ITC79 | Borgolavezzaro | NO | Piedmont | 2011 |
| ITC80 | Borgolavezzaro | NO | Piedmont | 2011 |
| ITC81 | Borgolavezzaro | NO | Piedmont | 2011 |
| ITC82 | Borgolavezzaro | NO | Piedmont | 2011 |
| ITC83 | Borgo Vercelli | VC | Piedmont | 2012 |
| ITC84 | Arborio | VC | Piedmont | 2012 |
| ITC85 | Morano Po | AL | Piedmont | 2012 |
| ITC86 | Borgolavezzaro | NO | Piedmont | 2012 |
| ITC87 | Noviglio | MI | Lombardy | 2012 |
| ITC88 | Castellazzo N.se | NO | Piedmont | 2012 |
| ITC89 | Vigevano | PV | Lombardy | 2012 |
| ITC90 | Morano Po | AL | Piedmont | 2012 |
| ITC91 | S. Giacomo V.se | VC | Piedmont | 2012 |
| ITC92 | Rovasenda | VC | Piedmont | 2012 |
| ITC93 | Borgolavezzaro | NO | Piedmont | 2012 |
| ITC94 | Trino | VC | Piedmont | 2012 |
| ITC95 | Trino | VC | Piedmont | 2012 |
| ITC96 | Arborio | VC | Piedmont | 2012 |
| ITC97 | S. Giacomo V.se | VC | Piedmont | 2012 |
| ITC98 | Rovasenda | VC | Piedmont | 2012 |
| ITC99 | Rovasenda | VC | Piedmont | 2012 |
| ITC100 | Lignana | VC | Piedmont | 2012 |
| ITC101 | Borgolavezzaro | NO | Piedmont | 2012 |
| ITC102 | Morano Po | AL | Piedmont | 2012 |
| ITC103 | S. Giacomo V.se | VC | Piedmont | 2012 |
| ITC104 | Rovasenda | VC | Piedmont | 2012 |
| ITC105 | Rovasenda | VC | Piedmont | 2012 |
| ITC106 | Borgolavezzaro | NO | Piedmont | 2012 |
| ITC107 | Casalbeltrame | NO | Piedmont | 2012 |
| ITC108 | Lignana | VC | Piedmont | 2012 |
| ITC109 | Trino | VC | Piedmont | 2012 |
| ITC110 | S. Giacomo V.se | VC | Piedmont | 2012 |
| ITC112 | Rovasenda | VC | Piedmont | 2012 |
| ITC113 | Lignana | VC | Piedmont | 2012 |
| ITC114 | Borgolavezzaro | NO | Piedmont | 2012 |
| ITC115 | Borgolavezzaro | NO | Piedmont | 2012 |
| ITC116 | Borgolavezzaro | NO | Piedmont | 2012 |
| ITC117 | Borgolavezzaro | NO | Piedmont | 2012 |
| ITC118 | Borgolavezzaro | NO | Piedmont | 2012 |
| ITC119 | Rovasenda | VC | Piedmont | 2012 |
| ITC120 | Borgolavezzaro | NO | Piedmont | 2012 |
| ITC121 | Casalbeltrame | NO | Piedmont | 2012 |
| ITC122 | Lignana | VC | Piedmont | 2012 |
| ITC123 | Trino | VC | Piedmont | 2012 |
| ITC124 | Trino | VC | Piedmont | 2012 |
| ITC125 | Arborio | VC | Piedmont | 2012 |
| ITC126 | S. Giacomo V.se | VC | Piedmont | 2012 |
| ITC127 | Rovasenda | VC | Piedmont | 2012 |
| ITC128 | Rovasenda | VC | Piedmont | 2012 |
| ITC129 | Lignana | VC | Piedmont | 2012 |
| ITC130 | Borgolavezzaro | NO | Piedmont | 2012 |
| ITC131 | Borgolavezzaro | NO | Piedmont | 2012 |
| ITC132 | Borgolavezzaro | NO | Piedmont | 2012 |
| ITC133 | Borgo Vercelli | VC | Piedmont | 2012 |
| ITC134 | Arborio | VC | Piedmont | 2012 |
| ITC135 | Morano Po | AL | Piedmont | 2012 |
| ITC136 | Rovasenda | VC | Piedmont | 2012 |
| ITC137 | Borgolavezzaro | NO | Piedmont | 2012 |
| ITC138 | Casalbeltrame | NO | Piedmont | 2012 |
| ITC140 | Lignana | VC | Piedmont | 2012 |
| ITC141 | Borgolavezzaro | NO | Piedmont | 2012 |
| ITC142 | Borgolavezzaro | NO | Piedmont | 2012 |
| ITC143 | Borgolavezzaro | NO | Piedmont | 2012 |
| ITC144 | Borgolavezzaro | NO | Piedmont | 2012 |
| ITC145 | Borgolavezzaro | NO | Piedmont | 2012 |
| ITC146 | Borgolavezzaro | NO | Piedmont | 2012 |
| ITC147 | Borgolavezzaro | NO | Piedmont | 2012 |
| ITC148 | Trino | VC | Piedmont | 2012 |
| ITC149 | Arborio | VC | Piedmont | 2012 |
| ITC150 | S. Giacomo V.se | VC | Piedmont | 2012 |
| ITC151 | Rovasenda | VC | Piedmont | 2012 |
| CRR1 | Castello d'Agogna | PV | Lombardy | 2020 |
| FMO1.1 | Roverbella | MN | Lombardy | 2020 |
| FMO2.2 | Erbè | VR | Veneto | 2020 |
| FMO3.3 | Mozzecane | VR | Veneto | 2020 |
| FMO4.2 | Codigoro | FE | Emilia-Romagna | 2020 |
| FMO5.1 | Codigoro | FE | Emilia-Romagna | 2020 |
| FMO6.2 | Jolanda di Savoia | FE | Emilia-Romagna | 2020 |
| NO1.1 | San Pietro Mosezzo | NO | Piedmont | 2020 |
| NO2.2 | San Pietro Mosezzo | NO | Piedmont | 2020 |
| NO3.3 | Galliate | NO | Piedmont | 2020 |
| NO4.2 | Vespolate | NO | Piedmont | 2020 |
| NO5.1 | Romentino | NO | Piedmont | 2020 |
| NO6.2 | Romentino | NO | Piedmont | 2020 |
| NO7.1 | San Pietro Mosezzo | NO | Piedmont | 2020 |
| PV1.2 | Cava Manara | PV | Lombardy | 2020 |
| PV2.2 | Zinasco | PV | Lombardy | 2020 |
| PV3.1 | Soltarico | PV | Lombardy | 2020 |
| PV4.2 | Rognano | PV | Lombardy | 2020 |
| PV5.2 | Zeccone | PV | Lombardy | 2020 |
| PV6.2 | Cassolnovo | PV | Lombardy | 2020 |
| PV7.1 | Vigevano | PV | Lombardy | 2020 |
| PV8.2 | Nicorvo | PV | Lombardy | 2020 |
| PV9.2 | Groppello Cairoli | PV | Lombardy | 2020 |
| PV10.3 | Giussago | PV | Lombardy | 2020 |
| PV11.2 | Gravellona Lomellina | PV | Lombardy | 2020 |
| VC1.3 | Carisio | VC | Piedmont | 2020 |
| VC2.3 | Carisio | VC | Piedmont | 2020 |
| VC3.1 | Carisio | VC | Piedmont | 2020 |
| VC4.2 | Lenta | VC | Piedmont | 2020 |
| VC5.1 | Trino | VC | Piedmont | 2020 |
| VC6.2 | San Giacomo Vercellese | VC | Piedmont | 2020 |
| VC7.2 | Casanova Elvo | VC | Piedmont | 2020 |
| BI1 | Gifflenga | BI | Piedmont | 2021 |
| BI2 | Salussola | BI | Piedmont | 2021 |
| FE3.a | Codigoro | FE | Emilia-Romagna | 2021 |
| LO1.b | Sant'Angelo Lodigiano | LO | Lombardy | 2021 |
| LOM4b | Tromello | PV | Lombardy | 2021 |
| LOM5 | Lomello | PV | Lombardy | 2021 |
| LOM6b | Pieve del Cairo | PV | Lombardy | 2021 |
| LOM8 | Cassolnovo | PV | Lombardy | 2021 |
| LOM12 | Castelnovetto | PV | Lombardy | 2021 |
| LOM16 | Mortara | PV | Lombardy | 2021 |
| LOM22 | Cozzo | PV | Lombardy | 2021 |
| MI2 | Gaggiano | MI | Lombardy | 2021 |
| NO3.A | S. Pietro Mosezzo | NO | Piedmont | 2021 |
| NO5 | San Nazzaro Sesia | NO | Piedmont | 2021 |
| NO6c | Novara | NO | Piedmont | 2021 |
| NO9a | Granozzo | NO | Piedmont | 2021 |
| OR1.b | Oristano | OR | Sardinia | 2021 |
| PV2 | Landriano | PV | Lombardy | 2021 |
| PV6 | Roncaro | PV | Lombardy | 2021 |
| PV8 | Albuzzano | PV | Lombardy | 2021 |
| VC1 | Buronzo | VC | Piedmont | 2021 |
| VC2 | Olcenengo | VC | Piedmont | 2021 |
| VC10 | Bianzè | VC | Piedmont | 2021 |
| VC12 | Tronzano | VC | Piedmont | 2021 |
| VC17 | Lignana | VC | Piedmont | 2021 |
| VC18 | Greggio | VC | Piedmont | 2021 |
| VC22.b | Crescentino | VC | Piedmont | 2021 |
| VR1.A | Isola della Scala | VR | Veneto | 2021 |
| CRR.F | Castello d'Agogna | PV | Lombardy | 2022 |
| FE4 | Riva del Po | FE | Emilia-Romagna | 2022 |
| LO3 | San Martino in Strada | LO | Lombardy | 2022 |
| LOM1 | Robbio | PV | Lombardy | 2022 |
| LOM3 | Zerbolò | PV | Lombardy | 2022 |
| LOM5 | Semiana | PV | Lombardy | 2022 |
| LOM7 | Dorno | PV | Lombardy | 2022 |
| LOM8 | Carbonara al Ticino | PV | Lombardy | 2022 |
| LOM13 | Vigevano | PV | Lombardy | 2022 |
| LOM14 | Cozzo | PV | Lombardy | 2022 |
| LOM15 | Gambolò | PV | Lombardy | 2022 |
| LOM19 | Olevano | PV | Lombardy | 2022 |
| MI2 | Rosate | MI | Lombardy | 2022 |
| MI4 | Rosate | MI | Lombardy | 2022 |
| MI5 | Casarile | MI | Lombardy | 2022 |
| MN1 | Castelberforte | MN | Lombardy | 2022 |
| MN2 | Bigarello | MN | Lombardy | 2022 |
| NO1 | Sillavengo | NO | Piedmont | 2022 |
| NO2 | Caltignaga | NO | Piedmont | 2022 |
| NO4a | Recetto | NO | Piedmont | 2022 |
| NO5 | Cameri | NO | Piedmont | 2022 |
| NO8 | Trecate | NO | Piedmont | 2022 |
| NO10 | Garbagna Novarese | NO | Piedmont | 2022 |
| NO13 | Vinzaglio | NO | Piedmont | 2022 |
| OR3 | Zerfaliu | OR | Sardinia | 2022 |
| PV6 | Sant' Alessio con Vialone | PV | Lombardy | 2022 |
| PV8 | Spessa Po | PV | Lombardy | 2022 |
| VC7 | Santhià | VC | Piedmont | 2022 |
| VC11 | Pezzana | VC | Piedmont | 2022 |
| VC23 | Morano Po | VC | Piedmont | 2022 |
| VC27 | Casanova Elvo | VC | Piedmont | 2022 |
| VR1 | Vigasio | VR | Veneto | 2022 |
| CM1 | Casale Monferrato | AL | Piedmont | 2022 |
| CR1 | Crova | VC | Piedmont | 2022 |
| RO1 | Rovasenda | VC | Piedmont | 2022 |
| MA2 | Massazza | BI | Piedmont | 2022 |

**Table S3.** Selected *P. oryzae* strains representative of the pathogen’s genetic diversity in Italy.

| Strain | Year of isolation | Geographical origin | TUCC code | Cluster |
| --- | --- | --- | --- | --- |
| NO4.2 | 2020 | Novara | TUCC00001089 | 2 |
| PV4.2 | 2020 | Pavia | TUCC00001090 | 1 |
| VC2.3 | 2020 | Vercelli | TUCC00001091 | 5 |
| LOM4b | 2021 | Pavia | TUCC00001092 | 3 |
| LOM5 | 2021 | Pavia | TUCC00001093 | 4 |
| LOM8 | 2021 | Pavia | TUCC00001094 | 5 |
| CR1 | 2022 | Vercelli | TUCC00001095 | 3 |
| MI4 | 2022 | Milano | TUCC00001096 | 2 |

**Table S4.** *P. oryzae* strains geographic origin, sampling year and cluster assignment.

| Strain | Year | Province | Region | Cluster |
| --- | --- | --- | --- | --- |
| ITC01 | 2011 | Vercelli | Piedmont | 5 |
| ITC02 | 2011 | Vercelli | Piedmont | 5 |
| ITC03 | 2011 | Pavia | Lombardy | 1 |
| ITC04 | 2011 | Novara | Piedmont | 1 |
| ITC05 | 2011 | Novara | Piedmont | 3 |
| ITC06 | 2011 | Novara | Piedmont | 3 |
| ITC07 | 2011 | Novara | Piedmont | 5 |
| ITC08 | 2011 | Novara | Piedmont | 5 |
| ITC10 | 2011 | Novara | Piedmont | 4 |
| ITC11 | 2011 | Novara | Piedmont | 5 |
| ITC12 | 2011 | Novara | Piedmont | 5 |
| ITC13 | 2011 | Novara | Piedmont | 5 |
| ITC14 | 2011 | Novara | Piedmont | 3 |
| ITC15 | 2011 | Novara | Piedmont | 5 |
| ITC16 | 2011 | Novara | Piedmont | 5 |
| ITC18 | 2011 | Novara | Piedmont | 3 |
| ITC20 | 2011 | Novara | Piedmont | 5 |
| ITC21 | 2011 | Novara | Piedmont | 3 |
| ITC22 | 2011 | Novara | Piedmont | 5 |
| ITC24 | 2011 | Novara | Piedmont | 5 |
| ITC25 | 2011 | Novara | Piedmont | 5 |
| ITC26 | 2011 | Novara | Piedmont | 5 |
| ITC27 | 2011 | Novara | Piedmont | 2 |
| ITC28 | 2011 | Novara | Piedmont | 5 |
| ITC29 | 2011 | Vercelli | Piedmont | 5 |
| ITC30 | 2011 | Vercelli | Piedmont | 5 |
| ITC31 | 2011 | Alessandria | Piedmont | 3 |
| ITC32 | 2011 | Novara | Piedmont | 1 |
| ITC34 | 2011 | Novara | Piedmont | 5 |
| ITC35 | 2011 | Pavia | Lombardy | 3 |
| ITC36 | 2011 | Alessandria | Piedmont | 1 |
| ITC38 | 2011 | Vercelli | Piedmont | 3 |
| ITC39 | 2011 | Novara | Piedmont | 4 |
| ITC40 | 2011 | Novara | Piedmont | 1 |
| ITC41 | 2011 | Novara | Piedmont | 4 |
| ITC42 | 2011 | Vercelli | Piedmont | 5 |
| ITC43 | 2011 | Novara | Piedmont | 4 |
| ITC44 | 2011 | Novara | Piedmont | 5 |
| ITC46 | 2011 | Novara | Piedmont | 4 |
| ITC48 | 2011 | Novara | Piedmont | 4 |
| ITC50 | 2011 | Novara | Piedmont | 4 |
| ITC51 | 2011 | Novara | Piedmont | 3 |
| ITC52 | 2011 | Novara | Piedmont | 1 |
| ITC53 | 2011 | Novara | Piedmont | 4 |
| ITC55 | 2011 | Novara | Piedmont | 1 |
| ITC57 | 2011 | Novara | Piedmont | 4 |
| ITC58 | 2011 | Novara | Piedmont | 4 |
| ITC60 | 2011 | Novara | Piedmont | 4 |
| ITC61 | 2011 | Novara | Piedmont | 5 |
| ITC62 | 2011 | Novara | Piedmont | 3 |
| ITC63 | 2011 | Novara | Piedmont | 4 |
| ITC65 | 2011 | Novara | Piedmont | 5 |
| ITC66 | 2011 | Novara | Piedmont | 3 |
| ITC67 | 2011 | Novara | Piedmont | 3 |
| ITC69 | 2011 | Novara | Piedmont | 5 |
| ITC71 | 2011 | Novara | Piedmont | 4 |
| ITC72 | 2011 | Novara | Piedmont | 5 |
| ITC74 | 2011 | Novara | Piedmont | 4 |
| ITC77 | 2011 | Novara | Piedmont | 1 |
| ITC81 | 2011 | Novara | Piedmont | 2 |
| ITC82 | 2011 | Novara | Piedmont | 1 |
| ITC83 | 2012 | Vercelli | Piedmont | 5 |
| ITC84 | 2012 | Vercelli | Piedmont | 1 |
| ITC85 | 2012 | Alessandria | Piedmont | 1 |
| ITC86 | 2012 | Novara | Piedmont | 5 |
| ITC87 | 2012 | Milano | Lombardy | 2 |
| ITC88 | 2012 | Novara | Piedmont | 3 |
| ITC89 | 2012 | Pavia | Lombardy | 3 |
| ITC90 | 2012 | Alessandria | Piedmont | 2 |
| ITC91 | 2012 | Vercelli | Piedmont | 5 |
| ITC92 | 2012 | Vercelli | Piedmont | 3 |
| ITC93 | 2012 | Novara | Piedmont | 5 |
| ITC94 | 2012 | Novara | Piedmont | 5 |
| ITC95 | 2012 | Novara | Piedmont | 2 |
| ITC96 | 2012 | Novara | Piedmont | 4 |
| ITC97 | 2012 | Novara | Piedmont | 1 |
| ITC99 | 2012 | Novara | Piedmont | 5 |
| ITC100 | 2012 | Novara | Piedmont | 5 |
| ITC101 | 2012 | Novara | Piedmont | 4 |
| ITC103 | 2012 | Vercelli | Piedmont | 5 |
| ITC104 | 2012 | Vercelli | Piedmont | 1 |
| ITC105 | 2012 | Vercelli | Piedmont | 5 |
| ITC106 | 2012 | Novara | Piedmont | 4 |
| ITC107 | 2012 | Novara | Piedmont | 5 |
| ITC108 | 2012 | Vercelli | Piedmont | 3 |
| ITC110 | 2012 | Novara | Piedmont | 5 |
| ITC112 | 2012 | Novara | Piedmont | 4 |
| ITC113 | 2012 | Novara | Piedmont | 5 |
| ITC114 | 2012 | Novara | Piedmont | 5 |
| ITC117 | 2012 | Novara | Piedmont | 3 |
| ITC118 | 2012 | Novara | Piedmont | 2 |
| ITC119 | 2012 | Vercelli | Piedmont | 2 |
| ITC120 | 2012 | Novara | Piedmont | 3 |
| ITC124 | 2012 | Novara | Piedmont | 5 |
| ITC126 | 2012 | Novara | Piedmont | 2 |
| ITC127 | 2012 | Novara | Piedmont | 5 |
| ITC128 | 2012 | Novara | Piedmont | 1 |
| ITC130 | 2012 | Novara | Piedmont | 3 |
| ITC131 | 2012 | Novara | Piedmont | 5 |
| ITC132 | 2012 | Novara | Piedmont | 2 |
| ITC133 | 2012 | Vercelli | Piedmont | 2 |
| ITC136 | 2012 | Vercelli | Piedmont | 2 |
| ITC137 | 2012 | Novara | Piedmont | 2 |
| ITC138 | 2012 | Novara | Piedmont | 5 |
| ITC141 | 2012 | Novara | Piedmont | 5 |
| ITC142 | 2012 | Novara | Piedmont | 1 |
| ITC143 | 2012 | Novara | Piedmont | 5 |
| ITC144 | 2012 | Novara | Piedmont | 3 |
| ITC146 | 2012 | Novara | Piedmont | 1 |
| ITC147 | 2012 | Novara | Piedmont | 5 |
| ITC149 | 2012 | Novara | Piedmont | 5 |
| ITC150 | 2012 | Novara | Piedmont | 5 |
| CRR1 | 2020 | Pavia | Lombardy | 5 |
| FMO1.1 | 2020 | Mantova | Lombardy | 2 |
| FMO2.2 | 2020 | Verona | Veneto | 5 |
| FMO3.3 | 2020 | Verona | Veneto | 5 |
| FMO4.2 | 2020 | Ferrara | Emilia-Romagna | 5 |
| FMO5.1 | 2020 | Ferrara | Emilia-Romagna | 3 |
| FMO6.2 | 2020 | Ferrara | Emilia-Romagna | 5 |
| NO1.1 | 2020 | Novara | Piedmont | 3 |
| NO3.3 | 2020 | Novara | Piedmont | 3 |
| NO4.2 | 2020 | Novara | Piedmont | 2 |
| NO6.2 | 2020 | Novara | Piedmont | 4 |
| NO7.1 | 2020 | Novara | Piedmont | 5 |
| PV1.2 | 2020 | Pavia | Lombardy | 2 |
| PV2.2 | 2020 | Pavia | Lombardy | 2 |
| PV3.1 | 2020 | Pavia | Lombardy | 2 |
| PV4.2 | 2020 | Pavia | Lombardy | 1 |
| PV5.2 | 2020 | Pavia | Lombardy | 4 |
| PV6.2 | 2020 | Pavia | Lombardy | 5 |
| PV7.1 | 2020 | Pavia | Lombardy | 2 |
| PV8.2 | 2020 | Pavia | Lombardy | 4 |
| PV9.2 | 2020 | Pavia | Lombardy | 5 |
| PV10.3 | 2020 | Pavia | Lombardy | 3 |
| VC1.3 | 2020 | Vercelli | Piedmont | 5 |
| VC2.3 | 2020 | Vercelli | Piedmont | 5 |
| VC3.1 | 2020 | Vercelli | Piedmont | 5 |
| VC4.2 | 2020 | Vercelli | Piedmont | 3 |
| VC5.1 | 2020 | Vercelli | Piedmont | 5 |
| VC6.2 | 2020 | Vercelli | Piedmont | 5 |
| VC7.2 | 2020 | Vercelli | Piedmont | 5 |
| BI1 | 2021 | Biella | Piedmont | 3 |
| BI2 | 2021 | Biella | Piedmont | 3 |
| FE3.a | 2021 | Ferrara | Emilia-Romagna | 3 |
| LO1.b | 2021 | Lodi | Lombardy | 2 |
| LOM4b | 2021 | Pavia | Lombardy | 3 |
| LOM5 | 2021 | Pavia | Lombardy | 4 |
| LOM6b | 2021 | Pavia | Lombardy | 3 |
| LOM8 | 2021 | Pavia | Lombardy | 5 |
| LOM12 | 2021 | Pavia | Lombardy | 5 |
| LOM22 | 2021 | Pavia | Lombardy | 2 |
| MI2 | 2021 | Milano | Lombardy | 2 |
| NO6c | 2021 | Novara | Piedmont | 3 |
| NO9a | 2021 | Novara | Piedmont | 5 |
| OR1.b | 2021 | Oristano | Sardinia | 5 |
| PV2 | 2021 | Pavia | Lombardy | 2 |
| PV6 | 2021 | Pavia | Lombardy | 5 |
| PV8 | 2021 | Pavia | Lombardy | 2 |
| VC1 | 2021 | Vercelli | Piedmont | 5 |
| VC2 | 2021 | Vercelli | Piedmont | 3 |
| VC10 | 2021 | Vercelli | Piedmont | 5 |
| VC12 | 2021 | Vercelli | Piedmont | 3 |
| VC17 | 2021 | Vercelli | Piedmont | 5 |
| VC18 | 2021 | Vercelli | Piedmont | 5 |
| VC22.b | 2021 | Vercelli | Piedmont | 5 |
| VR1.A | 2021 | Verona | Veneto | 5 |
| CRR.F | 2022 | Pavia | Lombardy | 3 |
| FE4 | 2022 | Ferrara | Emilia-Romagna | 4 |
| LO3 | 2022 | Lodi | Lombardy | 2 |
| LOM1 | 2022 | Pavia | Lombardy | 5 |
| LOM3 | 2022 | Pavia | Lombardy | 2 |
| LOM5 | 2022 | Pavia | Lombardy | 4 |
| LOM8 | 2022 | Pavia | Lombardy | 5 |
| LOM13 | 2022 | Pavia | Lombardy | 2 |
| LOM14 | 2022 | Pavia | Lombardy | 5 |
| LOM15 | 2022 | Pavia | Lombardy | 4 |
| LOM19 | 2022 | Pavia | Lombardy | 2 |
| MI2 | 2022 | Milano | Lombardy | 3 |
| MI4 | 2022 | Milano | Lombardy | 2 |
| MI5 | 2022 | Milano | Lombardy | 2 |
| MN1 | 2022 | Mantova | Lombardy | 2 |
| MN2 | 2022 | Mantova | Lombardy | 2 |
| NO1 | 2022 | Novara | Piedmont | 3 |
| NO2 | 2022 | Novara | Piedmont | 5 |
| NO4a | 2022 | Novara | Piedmont | 4 |
| NO5 | 2022 | Novara | Piedmont | 5 |
| NO8 | 2022 | Novara | Piedmont | 5 |
| NO10 | 2022 | Novara | Piedmont | 4 |
| NO13 | 2022 | Novara | Piedmont | 5 |
| OR3 | 2022 | Oristano | Sardinia | 5 |
| PV8 | 2022 | Pavia | Lombardy | 2 |
| VC7 | 2022 | Vercelli | Piedmont | 3 |
| VC11 | 2022 | Vercelli | Piedmont | 5 |
| VC23 | 2022 | Vercelli | Piedmont | 4 |
| VC27 | 2022 | Vercelli | Piedmont | 4 |
| VR1 | 2022 | Verona | Veneto | 2 |
| CM1 | 2022 | Alessandria | Piedmont | 3 |
| CR1 | 2022 | Vercelli | Piedmont | 3 |
| RO1 | 2022 | Vercelli | Piedmont | 4 |
| MA2 | 2022 | Biella | Piedmont | 4 |

**Table S5.** Absolute frequence of DAPC-inferred clusters based on sampling year and geographic origin. Total number of isolates assigned to each cluster for each region, as well as total number of isolates from each province for each sampling year, is also reported.

| Region | Province | | Year | Cluster 1 | Cluster 2 | Cluster 3 | Cluster 4 | Cluster 5 | Total |
| --- | --- | --- | --- | --- | --- | --- | --- | --- | --- |
|  |  | |  |  |  |  |  |  |  |
| Piedmont | Alessandria | | 2011 | 1 |  | 1 |  |  | 2 |
|  |  |  | 2012 | 1 | 1 |  |  |  | 2 |
|  |  |  | 2020 |  |  |  |  |  |  |
|  |  |  | 2021 |  |  |  |  |  |  |
|  |  |  | 2022 |  |  | 1 |  |  | 1 |
|  | Biella | | 2011 |  |  |  |  |  |  |
|  |  |  | 2012 |  |  |  |  |  |  |
|  |  |  | 2020 |  |  |  |  |  |  |
|  |  |  | 2021 |  |  | 2 |  |  | 2 |
|  |  |  | 2022 |  |  |  | 1 |  | 1 |
|  | Novara | | 2011 | 7 | 2 | 9 | 14 | 19 | 51 |
|  |  |  | 2012 | 4 | 5 | 5 | 4 | 18 | 36 |
|  |  |  | 2020 |  | 1 | 2 | 1 | 1 | 5 |
|  |  |  | 2021 |  |  | 1 |  | 1 | 2 |
|  |  |  | 2022 |  |  | 1 | 2 | 4 | 7 |
|  | Vercelli | | 2011 |  |  | 1 |  | 5 | 6 |
|  |  |  | 2012 | 2 | 3 | 2 |  | 4 | 11 |
|  |  |  | 2020 |  |  | 1 |  | 6 | 7 |
|  |  |  | 2021 |  |  | 2 |  | 5 | 7 |
|  |  |  | 2022 |  |  | 2 | 3 | 1 | 6 |
|  | Total | | | 15 | 12 | 30 | 25 | 64 | 146 |
|  |  | |  |  |  |  |  |  |  |
| Lombardy | Lodi | | 2011 |  |  |  |  |  |  |
|  |  |  | 2012 |  |  |  |  |  |  |
|  |  |  | 2020 |  |  |  |  |  |  |
|  |  |  | 2021 |  | 1 |  |  |  | 1 |
|  |  |  | 2022 |  | 1 |  |  |  | 1 |
|  | Mantova | | 2011 |  |  |  |  |  |  |
|  |  |  | 2012 |  |  |  |  |  |  |
|  |  |  | 2020 |  | 1 |  |  |  | 1 |
|  |  |  | 2021 |  |  |  |  |  |  |
|  |  |  | 2022 |  | 2 |  |  |  | 2 |
|  | Milano | | 2011 |  |  |  |  |  |  |
|  |  |  | 2012 |  | 1 |  |  |  | 1 |
|  |  |  | 2020 |  |  |  |  |  |  |
|  |  |  | 2021 |  | 1 |  |  |  | 1 |
|  |  |  | 2022 |  | 2 | 1 |  |  | 3 |
|  |  | |  |  |  |  |  |  |  |
|  | Pavia | | 2011 | 1 |  | 1 |  |  | 2 |
|  |  |  | 2012 |  |  | 1 |  |  | 1 |
|  |  |  | 2020 | 1 | 4 | 1 | 2 | 3 | 11 |
|  |  |  | 2021 |  | 3 | 2 | 1 | 3 | 9 |
|  |  |  | 2022 |  | 4 | 1 | 2 | 3 | 1 |
|  | Total | | | 2 | 20 | 7 | 5 | 9 | 34 |
|  |  | |  |  |  |  |  |  |  |
| Emilia-Romagna | Ferrara | | 2011 |  |  |  |  |  |  |
|  |  |  | 2012 |  |  |  |  |  |  |
|  |  |  | 2020 |  |  | 1 |  | 2 | 3 |
|  |  |  | 2021 |  |  | 1 |  |  | 1 |
|  |  |  | 2022 |  |  |  | 1 |  | 1 |
|  | Total | | | 0 | 0 | 2 | 1 | 2 | 5 |
|  |  | |  |  |  |  |  |  |  |
| Sardinia | Oristano | | 2011 |  |  |  |  |  |  |
|  |  |  | 2012 |  |  |  |  |  |  |
|  |  |  | 2020 |  |  |  |  |  |  |
|  |  |  | 2021 |  |  |  |  | 1 | 1 |
|  |  |  | 2022 |  |  |  |  | 1 | 1 |
|  | Total | | | 0 | 0 | 0 | 0 | 2 | 2 |
|  |  | |  |  |  |  |  |  |  |
| Veneto | Verona | | 2011 |  |  |  |  |  |  |
|  |  |  | 2012 |  |  |  |  |  |  |
|  |  |  | 2020 |  |  |  |  | 2 | 2 |
|  |  |  | 2021 |  |  |  |  | 1 | 1 |
|  |  |  | 2022 |  | 1 |  |  |  | 1 |
|  | Total | | | 0 | 1 | 0 | 0 | 3 | 4 |
|  |  |  | |  |  |  |  |  |  |


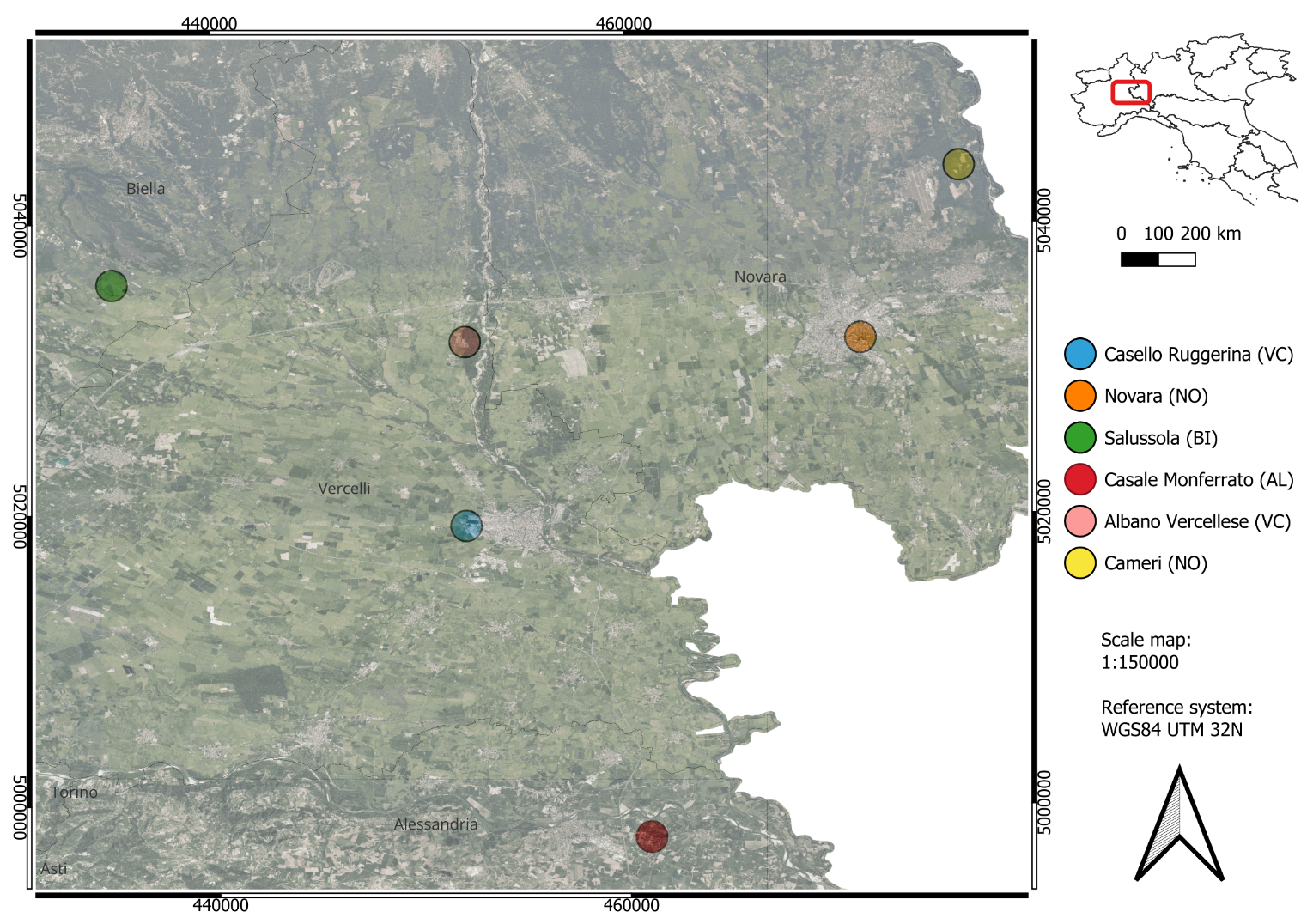


**Figure S1.** Location of Piedmontese weather stations considered for the correlation analysis. The map was made with QGIS Prizren v. 3.34.14.


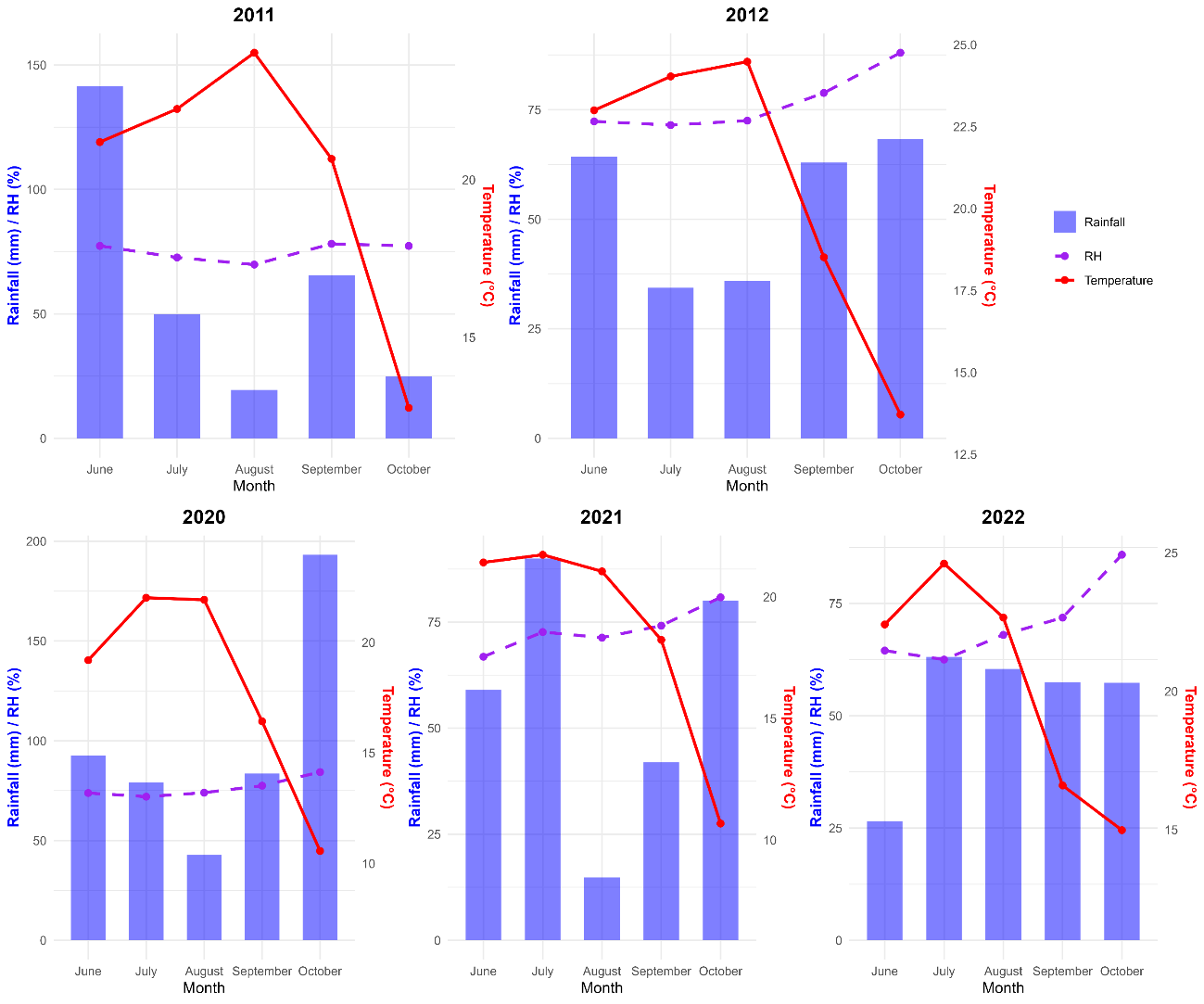


**Figure S2.** Monthly average values of rainfall (mm), relative humidity (%) and temperature (°C), collected from six Piedmontese weather stations, for each *P. oryzae* sampling year.


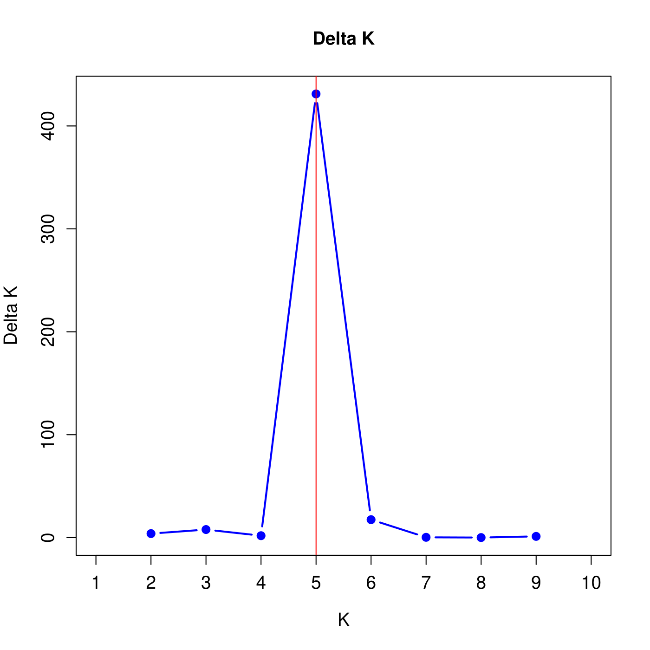


**b**


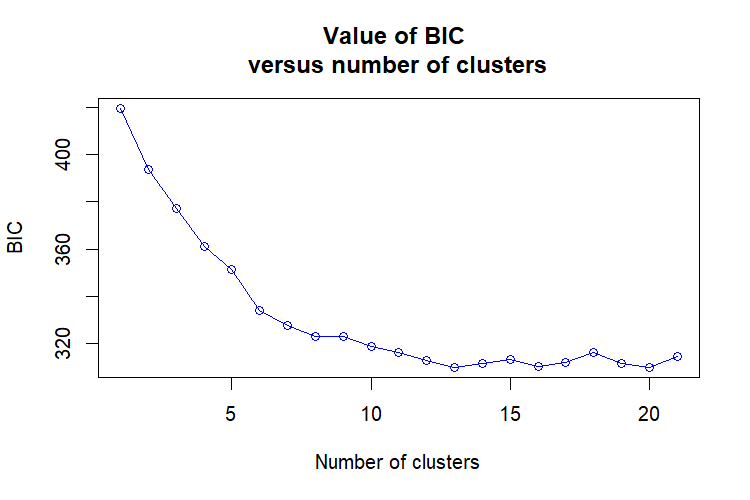


**a**

**Figure S3.** Estimation of the optimal number of genetic clusters by evaluating the BIC curve as a function of the number of K in the DAPC analysis (a) and through the Evanno ΔK statistical method (b). Both analyses led to choose 5 as the optimal number of K due to the “elbow” in the BIC curve corresponding to this value, as well as by observing a peak in the ΔK curve at K = 5.


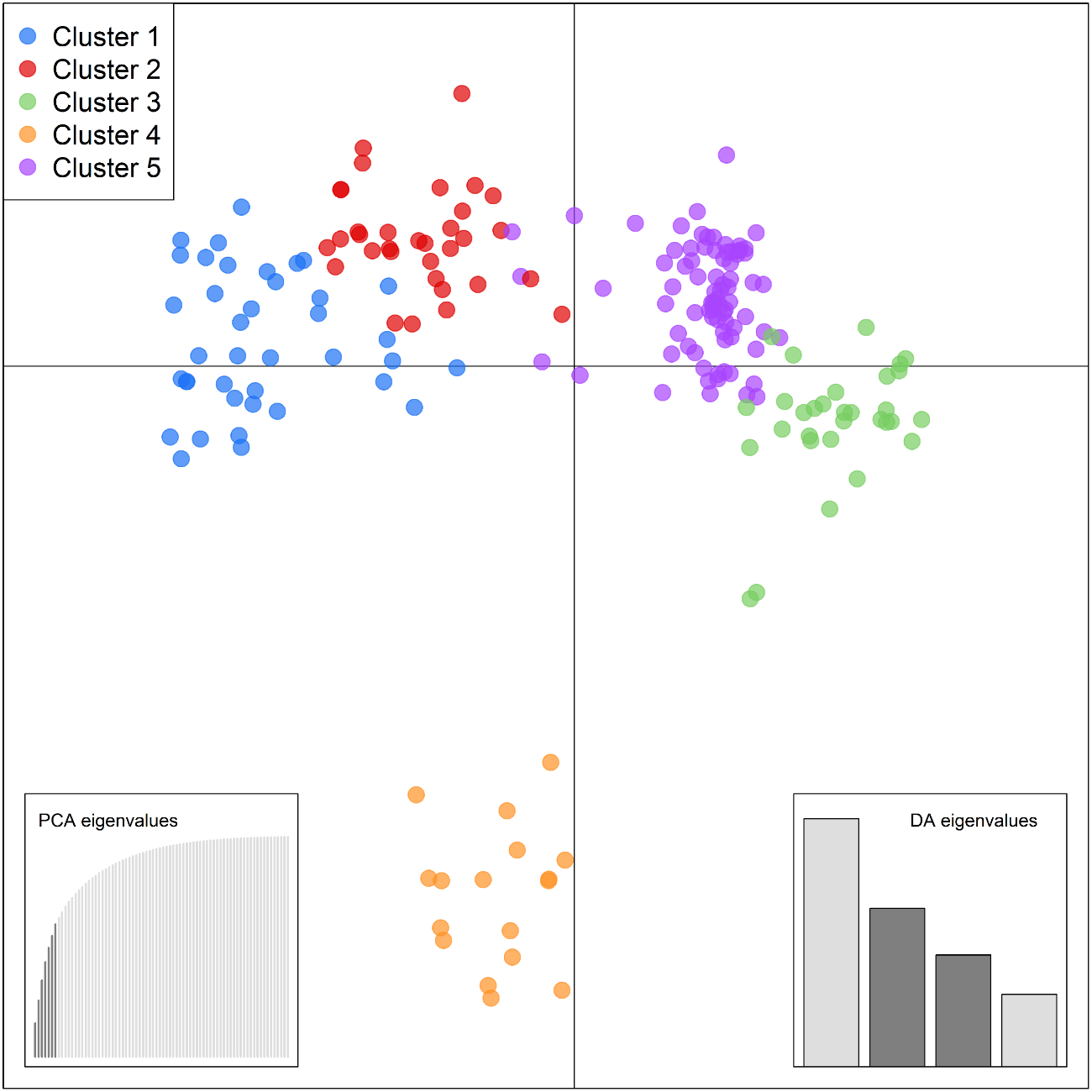


**Figure S4.** Projection of the DAPC analysis of the Italian *P. oryzae* population on axes 2 and 3.


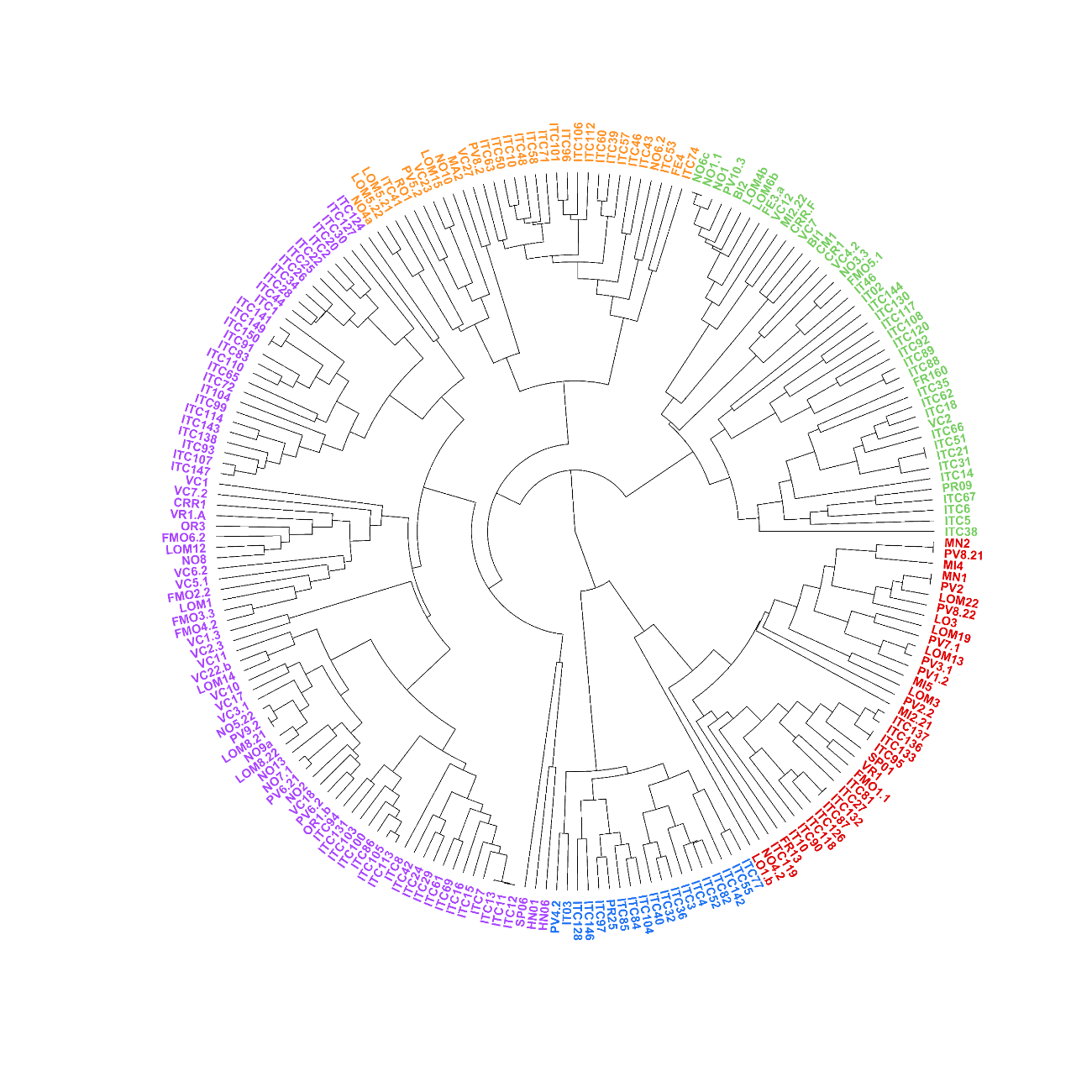

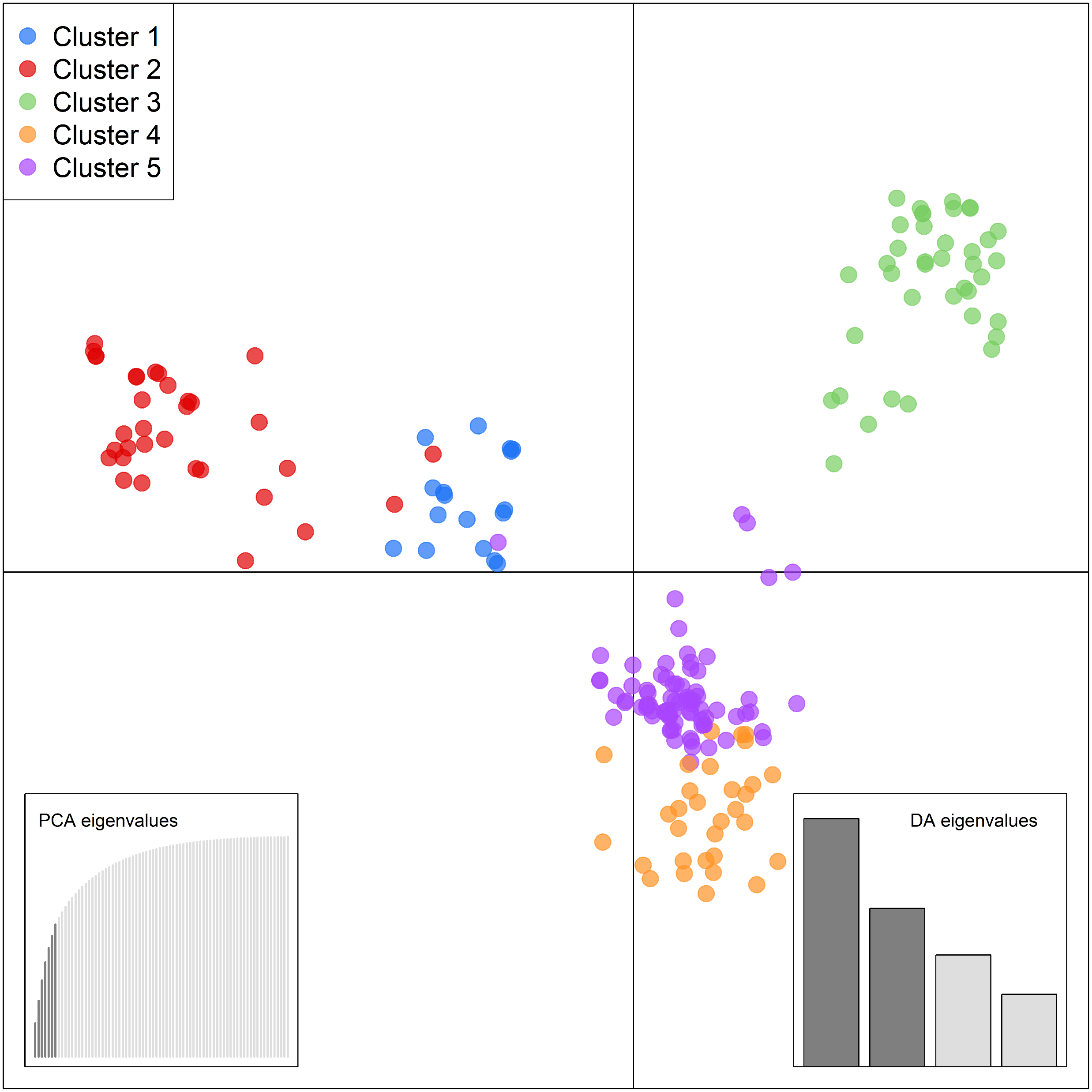


**Figure S5.** UPGMA dendrogram based on Nei’s genetic distance illustrating the cluster assignments of representative strains of European *P. oryzae* diversity from Roumen et al. (1997) and Thierry et al. (2022), indicated by arrows coloured based on DAPC cluster assignments, within the Italian *P. oryzae* population sampled in the present work.


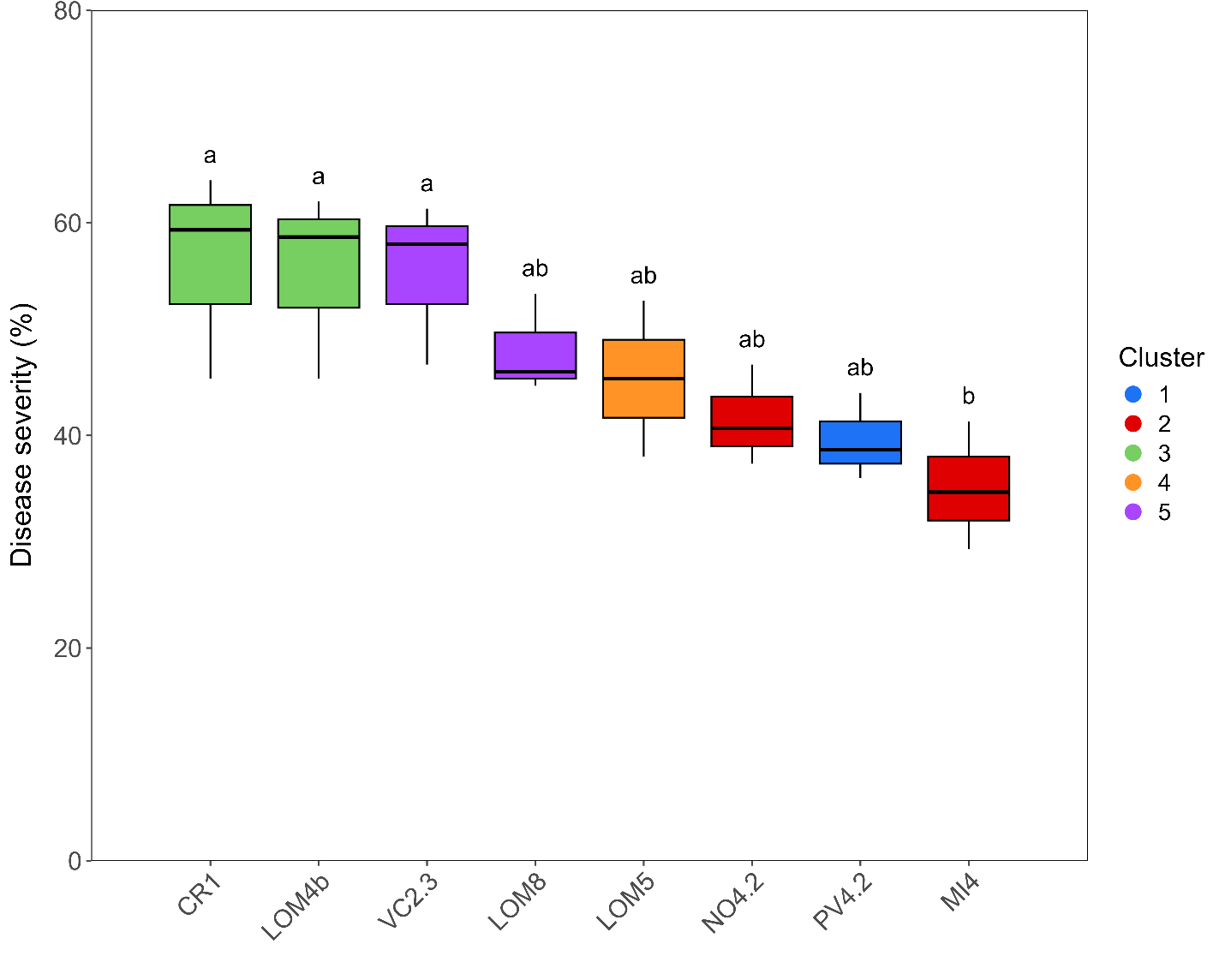


**Figure S6.** Virulence of eight *P. oryzae* strains, representative of Italian genetic diversity, evaluated as disease severity on leaves of 3 weeks old rice plants cultivar Vialone Nano Disease severity was scored using a scale with values from 1, absence of leaf blast lesions, to 5, extended sporulating lesions, according to Faivre-Rampant et al. (2011). The obtained scores were subsequently converted into percentage values as described in McKinney (1923).
